# The clinical utility of integrative genomics in childhood cancer extends beyond targetable mutations

**DOI:** 10.1038/s43018-022-00474-y

**Published:** 2022-12-30

**Authors:** Anita Villani, Scott Davidson, Nisha Kanwar, Winnie W. Lo, Yisu Li, Sarah Cohen-Gogo, Fabio Fuligni, Lisa-Monique Edward, Nicholas Light, Mehdi Layeghifard, Ricardo Harripaul, Larissa Waldman, Bailey Gallinger, Federico Comitani, Ledia Brunga, Reid Hayes, Nathaniel D. Anderson, Arun K. Ramani, Kyoko E. Yuki, Sasha Blay, Brittney Johnstone, Cara Inglese, Rawan Hammad, Catherine Goudie, Andrew Shuen, Jonathan D. Wasserman, Rosemarie E. Venier, Marianne Eliou, Miranda Lorenti, Carol Ann Ryan, Michael Braga, Meagan Gloven-Brown, Jianan Han, Maria Montero, Famida Spatare, James A. Whitlock, Stephen W. Scherer, Kathy Chun, Martin J. Somerville, Cynthia Hawkins, Mohamed Abdelhaleem, Vijay Ramaswamy, Gino R. Somers, Lianna Kyriakopoulou, Johann Hitzler, Mary Shago, Daniel A. Morgenstern, Uri Tabori, Stephen Meyn, Meredith S. Irwin, David Malkin, Adam Shlien

**Affiliations:** 1 Division of Hematology/Oncology, The Hospital for Sick Children, Toronto, Ontario Canada; 2 Department of Pediatrics, University of Toronto, Toronto, Ontario Canada; 3 Genetics and Genome Biology, The Hospital for Sick Children Research Institute, Toronto, Ontario Canada; 4 Department of Pediatric Laboratory Medicine, The Hospital for Sick Children, Toronto, Ontario Canada; 5 Institute of Medical Science, University of Toronto, Toronto, Ontario Canada; 6 Department of Molecular Genetics, University of Toronto, Toronto, Ontario Canada; 7 Cancer Genetics and High-Risk Program, Sunnybrook Health Sciences Centre, Toronto, Ontario Canada; 8 Department of Genetic Counselling, University of Toronto, Toronto, Ontario Canada; 9 Division of Clinical and Metabolic Genetics, The Hospital for Sick Children, Toronto, Ontario Canada; 10 Center for Computational Medicine, The Hospital for Sick Children, Toronto, Ontario Canada; 11 Division of Hematology, Faculty of Medicine, King Abdulaziz University, Jeddah, Saudi Arabia; 12 Division of Hematology-Oncology, McGill University Health Centre, Montreal, Quebec Canada; 13 Department of Pediatrics, McGill University, Montreal, Quebec Canada; 14 Division of Endocrinology, The Hospital for Sick Children, Toronto, Ontario Canada; 15 McLaughlin Centre, University of Toronto, Toronto, Ontario Canada; 16 Department of Laboratory Medicine and Pathobiology, University of Toronto, Toronto, Ontario Canada; 17 Developmental and Stem Cell Biology, The Hospital for Sick Children Research Institute, Toronto, Ontario Canada; 18 Center for Human Genomics and Precision Medicine, University of Wisconsin School of Medicine and Public Health, Madison, WI USA

**Keywords:** Cancer genomics, Paediatric cancer, Cancer

## Abstract

We conducted integrative somatic–germline analyses by deeply sequencing 864 cancer-associated genes, complete genomes and transcriptomes for 300 mostly previously treated children and adolescents/young adults with cancer of poor prognosis or with rare tumors enrolled in the SickKids Cancer Sequencing (KiCS) program. Clinically actionable variants were identified in 56% of patients. Improved diagnostic accuracy led to modified management in a subset. Therapeutically targetable variants (54% of patients) were of unanticipated timing and type, with over 20% derived from the germline. Corroborating mutational signatures (SBS3/BRCAness) in patients with germline homologous recombination defects demonstrates the potential utility of PARP inhibitors. Mutational burden was significantly elevated in 9% of patients. Sequential sampling identified changes in therapeutically targetable drivers in over one-third of patients, suggesting benefit from rebiopsy for genomic analysis at the time of relapse. Comprehensive cancer genomic profiling is useful at multiple points in the care trajectory for children and adolescents/young adults with cancer, supporting its integration into early clinical management.

## Abstract (web summary)

Shlien and colleagues report on 300 patients from the SickKids Cancer Sequencing program and identify clinically actionable variants in 56% of the patients by profiling somatic and germline data at multiple clinical time points.

## Main

Whereas the long-term survival for young people with cancer now approaches 85%, that for patients with relapsed, metastatic or treatment-refractory disease has remained dismal, with virtually no improvement in more than four decades across most disease subtypes^1^. The incorporation of research-based next-generation sequencing (NGS) technologies, including whole-genome sequencing (WGS) and transcriptome sequencing as well as targeted cancer gene or expression panels, to expand treatment options through identification of clinically actionable targets, offers promise to patients with ‘hard-to-cure’ cancers^2–6^. Furthermore, NGS offers an unprecedented opportunity to more deeply understand the long tail of rare pediatric tumors, for which there is currently limited genomic knowledge and no established effective treatment. The recognized potential for incorporation of NGS in the toolbox of diagnostic tests in oncology remains tempered by (1) reluctance to offer NGS testing early; (2) reluctance to rebiopsy in the context of relapse, metastatic or refractory disease; and (3) limited attention to the implications of germline events.

The genetic profile of childhood cancers has typically been characterized as ‘quiet’ because these cancers harbor a low overall burden of somatic substitution mutations at diagnosis; however, copy number changes and structural variants are more prevalent^2^. It is not known whether sporadic pediatric tumors can acquire sufficient mutations at relapse to become hypermutant—above the thresholds defined for adult cancers—and thereby be candidates for immune checkpoint inhibitor therapy.

Many mutations in childhood cancers arise years before diagnosis, having been either inherited from a parent or acquired postzygotically in early embryonic cells^7–11^. This suggests an opportunity for early intervention or therapeutic strategies that target the tumor’s evolutionary root. However, which patients and/or tumor types may benefit remains unclear. Mutations in *BRCA1* and other homologous recombination (HR) pathway components provide a compelling example. These mutations have been reported in the germline of patients with childhood cancer^3,4,7^; however, whether they are drivers or mere bystanders (as is the case in many adult tumors^12^) is unknown.

The SickKids Cancer Sequencing (KiCS) program is a prospective study of a demographically diverse population of children and adolescents/young adults (AYA) with refractory, metastatic, relapsed or rare cancers, as well as children with unresolved suspicion for cancer predisposition. In addition to establishing the clinical feasibility and utility of integrative tumor–germline sequencing in upfront diagnostics/prognostication and in therapeutic target identification, we sought to evaluate pediatric tumor etiology and evolution. We investigated the role of germline pathogenic variants in HR pathway genes as drivers of pediatric/AYA cancers and the change in mutational burden and tumor drivers over the course of the disease trajectory, to establish the value of rebiopsy at relapse. On the basis of our observations, we suggest that comprehensive somatic–germline genomic profiling at multiple time points is essential to understanding tumor evolution and impacts clinical management.

## Results

### Sequencing is feasible across a broad range of cancers

**Fig. 1:**
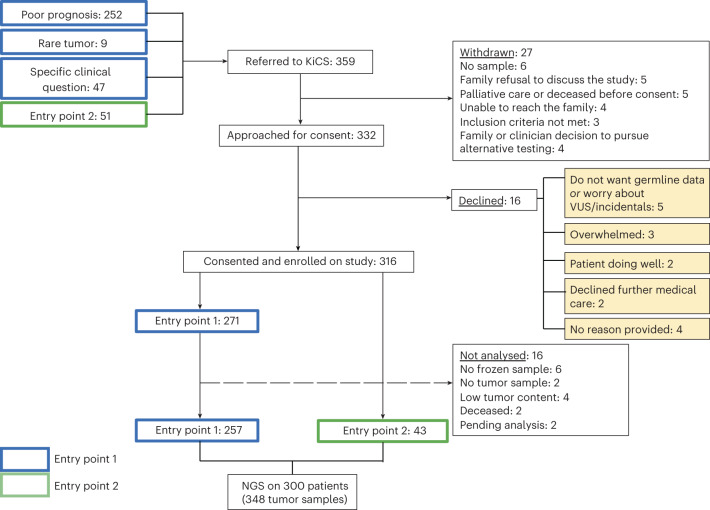
Overview of the KiCS study cohort and enrollment outcomes of referred patients. Entry Point 2: Suspicion for cancer predisposition syndrome. VUS, variants of uncertain significance.

**Fig. 2:**
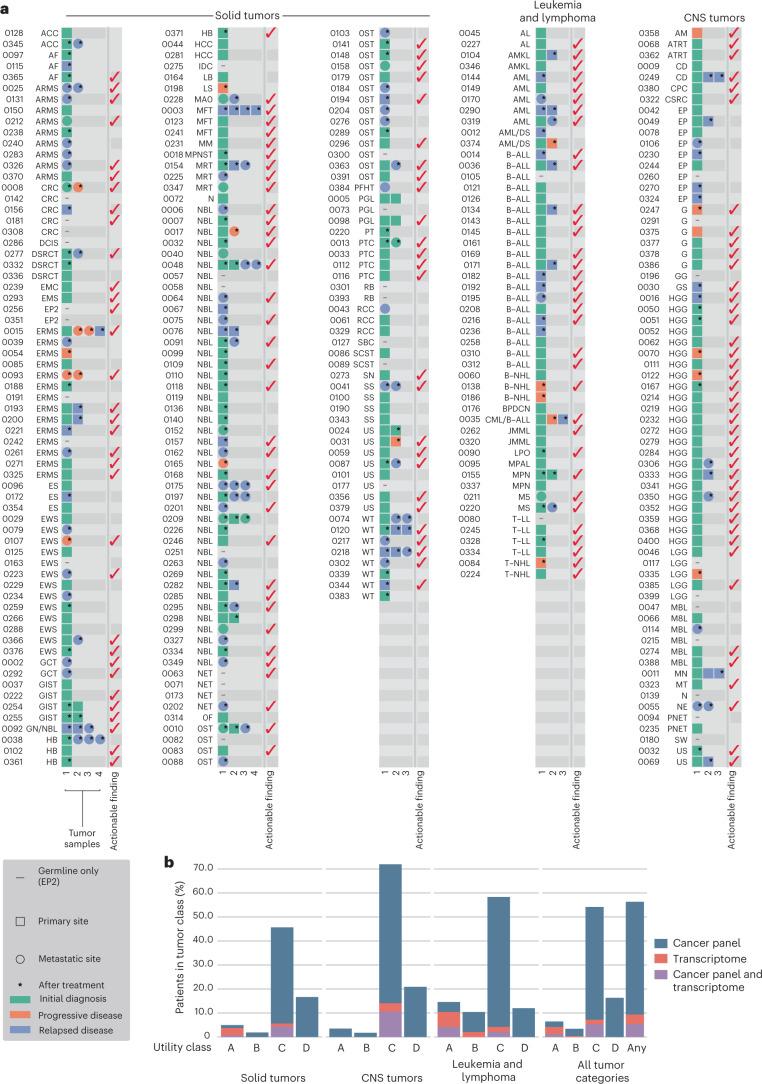
The KiCS study cohort: tumor and sample characteristics and summary of actionable findings. **a**, Each row, labeled by study ID and diagnosis (based on pathology report), corresponds to a study participant. The first four columns describe the tumor samples for each patient. Samples are arranged chronologically from left to right. Color indicates the disease state: green, initial diagnosis; blue, relapse; orange, progressive disease. Squares correspond to samples from the primary tumor site, and circles represent samples of metastatic sites. A star indicates a sample collected after the patient had received cancer-directed therapy. EP2 patients with multiple prior malignancies were classified according to their most recent tumor diagnosis. EP2 participants with no cancer diagnosis are coded as ‘EP2’ and are denoted with a gray dash if there was no accompanying tumor sample. The last column indicates participants with at least one actionable finding (red checkmark). Please see Supplementary Table 1 for a full list of tumor types and acronyms, categories of actionable findings and additional demographic information. *n* = 300 participants. Three participants (KiCS 32, 220 and 334) each had two primary tumors and are each represented twice. **b**, Frequency of actionable findings, by class of clinical utility. The height of each histogram is the percentage of patients with at least one actionable finding in that category. Patients with an actionable variant having more than one aspect of utility are recorded in each relevant category but are only counted once in the ‘any’ clinical utility class. The colors within each histogram represent the proportion of variants in that class detected by each NGS technology. For the A, B and C categories, percentages were calculated on the basis of a denominator of *n* = 264 (participants with somatic analysis). For the D and ‘any’ categories, percentages were calculated on the basis of a denominator of *n* = 300 total study participants. Source data

**Extended Data Fig. 1:**
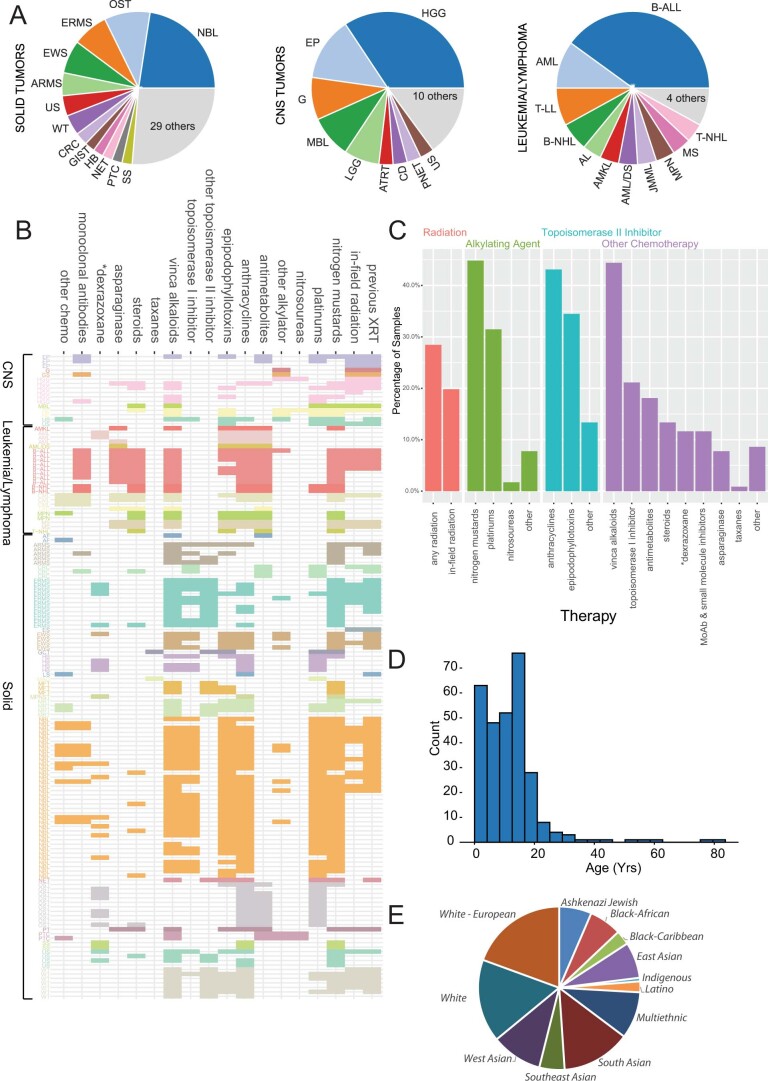
KiCS cohort characteristics. **(a)** Distribution of tumor types (n = 303; 3 participants had two independent primary tumors. N = 186 for solid tumors, n = 67 for CNS tumors, n = 50 for leukemia/lymphomas). *OTHER (Solid Tumors):* ACC, AF, DCIS, DSRCT, EMC, EMS, ES, GCT, HCC, IDC, LB, LS, MAO, MFT, MM, MPNST, MRT, N, OF, PFHT, PGL, PT, RB, RCC, SBC, SCST, SN. *OTHER (CNS Tumors):* AM, CPC, CSRC, GG, GS, MN, MT, N, NE, SW. *OTHER (Leukemia/Lymphoma):* BPDCN, CML, LPD, MPAL. Please see Supplementary Table S1 for a full list of tumor types and acronyms. (**b**, **c)** Representative example of prior therapeutic exposures of sequenced samples (N = 232 tumor samples from 176 patients). (**d)** Age distribution (N = 300 participants). (**e)** Ethnicity distribution (based on self-reported ethnicity provided by 41% of study participants, N = 123 participants (59% missing data)). Source data

**Extended Data Fig. 2:**
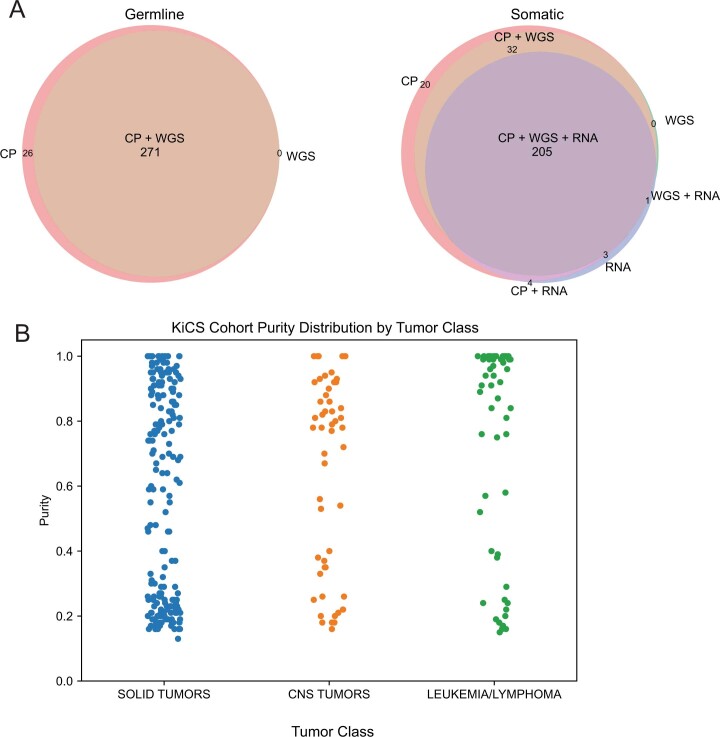
Sample sequencing and purity. **(a)** Venn diagram of sequencing technologies used. The proportion of patients whose tumors were analyzed using each combination of cancer-panel (CP -pink), whole-genome (WGS – green) and whole-transcriptome (WTS –violet) sequencing, and the proportion of patients whose germline DNA was analyzed using each combination of CP and WGS. (**b)** KiCS cohort purity distribution by tumor class. N = 292 tumor samples. Greater than 75% of samples have a purity of over 25%. Source data

From 29 April 2016 to 14 January 2020, 359 patients were referred to the KiCS program, comprising 252 patients with hard-to-cure tumors, 9 patients with rare tumors, 47 patients for whom specific clinical questions were posed (collectively entry point 1, EP1) and 51 patients suspected of cancer predisposition (entry point 2, EP2; Fig. 1). Five percent (16/332) of individuals declined participation following an informed consent discussion. The feasibility of sequencing pediatric and young adult tumors was high: only 18 individuals were not enrolled or were subsequently removed from the study owing to inadequate tumor material whereas 123 tumor specimens were retrieved from small biopsy samples. Ninety-five percent of samples were available in fresh frozen form while 19 formalin-fixed, paraffin-embedded (FFPE) tumor samples were included. The analyses reported here were based on the first 300 KiCS participants who underwent comprehensive sequencing, representing a wide spectrum of pediatric and young adult cancers. Fifty-six participants had more than one tumor sample analyzed, and 7 EP2 patients had tumor analysis as well, leading to a total of 348 tumor samples from 264 patients. Participant and tumor characteristics, as well as sample numbers, are outlined in Fig. 2a, Extended Data Fig. 1 and Supplementary Table 1. Three patients each had two histologically distinct primary tumors analyzed, but a denominator of 300 was used for clarity. Extended Data Fig. 2a details the proportion of participants’ samples analyzed using each sequencing platform, and Supplementary Table 2 summarizes sample-level sequencing metrics. The median age at enrollment of participants undergoing tumor–germline analysis was 7.1 years. Approximately 44% of these patients (41% of samples) had progressive or relapsed disease, and 58% of patients (57% of samples) had been exposed to chemotherapy and/or radiation therapy.

### Integrative analysis identifies multiple classes of actionable variants

In total, 56% of participants had at least one clinically actionable finding from comprehensive sequencing; 30.7%, 14.9% and 10.9% of participants had one, two or more than two findings, respectively (Fig. 2a,b). Category A variants, refining an unclear diagnosis, were documented in 17 participants who underwent somatic analysis (6%), with an impact on therapy in 14 of 17 cases (Table 1). In addition, our analysis was 100% concordant with standard clinical and cytogenetic testing for diagnostic/prognostic variants and fusions (for those with RNA available) in solid tumors, central nervous system (CNS) tumors and leukemias (Supplementary Table 3a). For an additional nine participants, our analysis provided further characterization of previous clinical findings (Supplementary Table 3b). Nine participants had sequencing findings that were of potential prognostic relevance (category B; Supplementary Table 4).

**Table 1:** Category A variants: actionable findings resulting in change or refinement of diagnosis

	Standard clinical analysis	KiCS/NGS analysis		
KiCS ID	Initial pathologic diagnosis and results from relevant clinical testing	Refined diagnosis based on KiCS analysis	Actionable finding	Impact
**Solid tumors**				
59	8805/3—undifferentiated sarcoma (M^1^, C^2^)	*BCOR*-fused sarcoma	*BCOR-CCNB3* fusion	No change in therapy
87	8805/3—undifferentiated sarcoma vs. 9150/3—hemangiopericytoma, malignant (C^3^)	*BCOR*-fused sarcoma	*BCOR-CCNB3* fusion	Changed to Ewing sarcoma-like therapy
141	9180/3—osteosarcoma, NOS (C40._, C41._)	*BCOR*-fused sarcoma	*BCOR-CCNB3* fusion	At relapse following osteosarcoma therapy, treated according to refined diagnosis with Ewing sarcoma-like therapy
239	Malignant small round blue cell tumor with *EWSR1* gene rearrangement (C^4^, M^5^)	8562/3—epithelial–myoepithelial carcinoma	*EWSR1-ZNF444* fusion	Selection of appropriate chemotherapy and improved prognostication
123	8825/1—myofibroblastic tumor, NOS (‘fibroblastic/myofibroblastic proliferation’) (I^6^, C^7^)	8821/1—aggressive fibromatosis/desmoid tumor	CTNNB1 p.T41A	Selection of desmoid-tailored chemotherapy instead of morbid surgical resection
241	8825/1—myofibroblastic tumor, NOS (‘fibroblastic neoplasm with nuclear β-catenin immunoreactivity, favor desmoid-type fibromatosis’) (C^8^, M^9^, I^10^)	8821/1—aggressive fibromatosis/desmoid tumor	Somatic *APC* copy number loss, exons 5–22, with homozygous loss of exons 10–15	Selection of desmoid-tailored chemotherapy instead of morbid surgical resection
356	8805/3—undifferentiated sarcoma (C49.2) (C^11^, M^12^, I^13^)	*CIC*-fused sarcoma	*CIC-NUTM2A* fusion	No change (patient deceased)
379	8800/3—sarcoma, NOS (C76.3) (I^14^, M^15^)	Primitive myxoid mesenchymal tumor of infancy	BCOR p.*1722Lext*34; RNA-seq: ITD and *BCOR*-driven tumor expression cluster	Selection of appropriate chemotherapy and improved prognostication
**Leukemia and lymphoma**				
192	9812/3—B lymphoblastic leukemia/lymphoma with t(9;22)(q34;q11.2); *BCR*-*ABL1* (C^16^)	9836/3—precursor B cell lymphoblastic leukemia	*EWSR1-PBX3* fusion (no *BCR-ABL* fusion)	Change of therapy (taken off TKI)
104	Preliminary: melanotic neuroectodermal tumor of infancy Final: ‘pseudo-sarcomatous mass with infiltrates of a primitive hematolymphoid neoplasm with predominance of blasts of M7 acute megakaryoblastic leukemia features’ (F^17^, C^18^, M^19^, I^20^)	9910/3—acute megakaryoblastic leukemia	*RBM15-MKL1* fusion	Initially treated as melanotic neuroectodermal tumor of infancy; changed to AMKL therapy
155	9960/3—myeloproliferative neoplasm, NOS (M^21^, C^22^)	Myeloproliferative neoplasm with *ETV6-ABL1* rearrangement	*ETV6-ABL1* fusion	Initiation of TKI (eventual allogeneic transplantation)
227	9801/3—acute leukemia, NOS (F^23^, M^24^)	9837/3—T lymphoblastic leukemia/lymphoma	*SET-NUP214* fusion, PHF6 p.V268Tfs*5 and NOTCH1 p.N386S	T-ALL therapy after other failed induction regimens, with omission of steroids
262	9946/3—juvenile myelomonocytic leukemia (C42.1) vs. acute myeloid leukemia (C^25^, F^26^, M^27^)	9946/3—juvenile myelomonocytic leukemia (C42.1)	KRAS p.G12A and monosomy 7	Confirmed decision to proceed to allogeneic hematopoietic stem cell transplantation
310	9836/3—precursor B cell lymphoblastic leukemia (C42.1) (F^28^, C^29^, M^30^)	9836/3—precursor B cell lymphoblastic leukemia (C42.1), Ph-like	SH2B3 p.F146Lfs*52; RNA-seq expression cluster: B-ALL, Ph-like, JAK–STAT	No change in therapy, but improved subclassification, prognostication and future treatment options
346	9910/3—acute megakaryoblastic leukemia (C42.1) (F^31^, C^32^)	9898/3—myeloid leukemia associated with Down syndrome	*GATA1* c.186_190delCTACA (p.Y62*)	Selection of appropriate chemotherapy (lower intensity for myeloid leukemia of Down syndrome, instead of high-intensity AML therapy)
**CNS tumors**				
70	9440/3—glioblastoma (C71._)	Low-grade glioma	FGFR1 p.K656M and germline NF1 p.S1468G	Initiation of targeted therapy instead of radiation therapy
323	8990/1—mesenchymal tumor (C71.0) (I^33^, C^34^, M^35^)	8824/0—myofibroma (C71.0)	PDGFRB p.P588delinsLP	Confirmed plan for no adjuvant therapy after resection, given benign entity

The original tumor diagnosis is presented along with the extent of clinical testing carried out, including cytogenetic (C), molecular (M), immunohistochemical (I) and flow cytometry (F) analyses. A refined diagnosis was suggested by cancer panel and/or RNA-seq findings, with the noted impact on clinical management. The results of clinical testing are indicated by the superscript numbers: (1) negative for *EWS-FLI1*, *CIC-DUX4* and *SSX-SYT* fusion transcripts; (2) negative for *SYT* and *EWS* gene rearrangements; (3) no malignant cells; (4) positive for *EWSR1* gene rearrangement; (5) negative for *EWS-WT1* (RT–PCR) and negative Nanostring assay for fusion transcripts; (6) β-catenin mostly cytoplasmic and perinuclear with possible focal nuclear staining; (7) negative for *FUS* gene rearrangement; (8) negative for *FUS*, *USPS* and *EWSR1* rearrangements; (9) negative Nanostring assay for fusion transcripts; (10) positive for β-catenin nuclear expression; (11) negative for *EWSR1* rearrangement; (12) failed analysis; (13) positive for CD99 membrane staining, WT1; negative for CKAE1/AE3, S100, SOX10, melanA, SMA, desmin, myogenin, TFE3, IN1 retained; (14) positive for vimentin and TLE-1, patchy staining for CD99, focal positivity for S100 and SOX0, BAF47 intact; negative for NB84, CD45, EMA, pan-keratin, myogenin, SMA, actin, caldesmon, CD34, CD31, GFAP, PLAP, glypican-3, WT-1, OCT4 and CD30; (15) negative Nanostring assay for fusion transcripts and negative Trusight RNA-seq for oncogenic fusion transcripts and mutations; (16) interpreted as being consistent with *BCR-ABL*; (17) negative for CD41 and CD61 (BMA); (18) negative for *MLL* rearrangement (BMA); (19) negative for t(1;22)(p13;q13)/*RBM15-MKL1* and negative sarcoma fusion panel (BMA); (20) negative for CD56, CD61 and factor VIII (BMBx), negative large panel and positive for CD43, CD61 and factor VIII (maxillary mass); (21) negative for *BCR-ABL* p210/p190, *PDGFRA-FIP1L1* and t(5;14)(q31;q32)/*IL3-IGH*, *JAK2* V617F, *JAK2* exon 12 mutations, *CALR*, *FLT3*-ITD, clonal rearrangements of TCR genes and *IGH* gene fusion; (22) negative for *FIP1L1-CHIC2-PDGFRA*, *PDGFRB* and *MYC*; (23) positive for CD34, CD2, CD7, CD33, CD38, CD11 and CD71 and negative for Tdt, MPO and cCD3; (24) negative for 29 recurrently mutated myeloid leukemia genes and positive for TCR rearrangement-γ-chain; (25) monosomy 7; (26) myeloblasts and population with monocytic differentiation; (27) negative RT–PCR for AML fusion transcripts and negative for *FLT3-*ITD; (28) consistent with precursor B lymphoblasts; (29) positive for iAMP21 and normal FISH analysis for *CRLF2*, *IGH*, *MYC*, *CDKN2A* and *TCF3*; (30) negative RT–PCR for canonical ALL fusion transcripts, positive LDA screen and negative Trusight RNA-seq for Ph-like fusion transcripts or mutations; (31) consistent with megakaryoblasts; (32) 49,XX,del(6)(q13q21),+8,+21,+21(20); (33) large panel; (34) negative for whole and segmental chromosome aberrations; (35) negative Nanostring assay for fusion transcripts and negative Trusight RNA-seq for oncogenic fusions and mutations. BMA, bone marrow aspirate; BMBx, bone marrow biopsy; LDA, low density array; TKI, tyrosine kinase inhibitor.

**Fig. 3:**
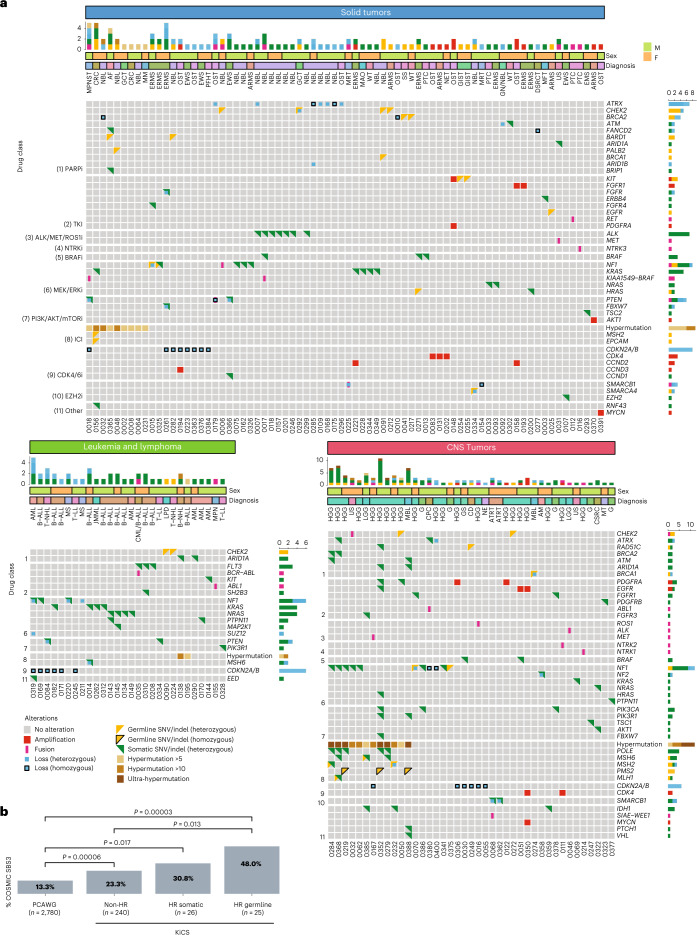
Oncoprint visualization of category C (therapeutic) clinically actionable findings and BRCAness in pediatric and AYA cancers in the KiCS cohort. **a**, Oncoprint visualization of the distribution of therapeutically actionable findings (category C). Findings are arranged in rows and grouped by the therapeutic agent indicated by each finding. Patients (*n* = 143; two patients with two primary tumors each represented twice) are arranged in columns. The top bar plot indicates the number of relevant mutations in each patient (that is, the number of variants that constitute therapeutic biomarkers in each patient). Some variants contribute together as a single actionable finding, for example, *PTEN* SNV and *PTEN* loss in KiCS 366 and *MSH2* germline and somatic SNVs, *POLE* SNV and ultra-hypermutation in KiCS 284. Variant details are depicted in Supplementary Table 5. The right-side bar plot depicts the number of patients harboring a finding. Red square, amplification; blue square, loss; pink vertical rectangle, fusion; yellow triangle, germline SNV/indel; green triangle, somatic SNV/indel; black border, homozygous mutation; brown square, hypermutation. Please see Supplementary Table 1 for a full list of tumor types and acronyms**. b**, The proportion of COSMIC single-substitution signature 3 (BRCAness mutational signature) in the PCAWG dataset compared to KiCS cohort patients with absence or presence of either somatic or germline variants affecting the HR pathway. The KiCS cohort is divided into three subsets based on the absence or presence of germline or somatic HRD. The proportion of samples with the SBS3 signature in each KiCS subset as well as the PCAWG dataset is shown by the height of the bars. Sample sizes (that is, the number of biologically independent samples) are shown on the *x* axis. Only statistically significant *P* values obtained by Fisher’s exact test (two sided) comparing each pair of datasets are shown. Source data

**Extended Data Fig. 3:**
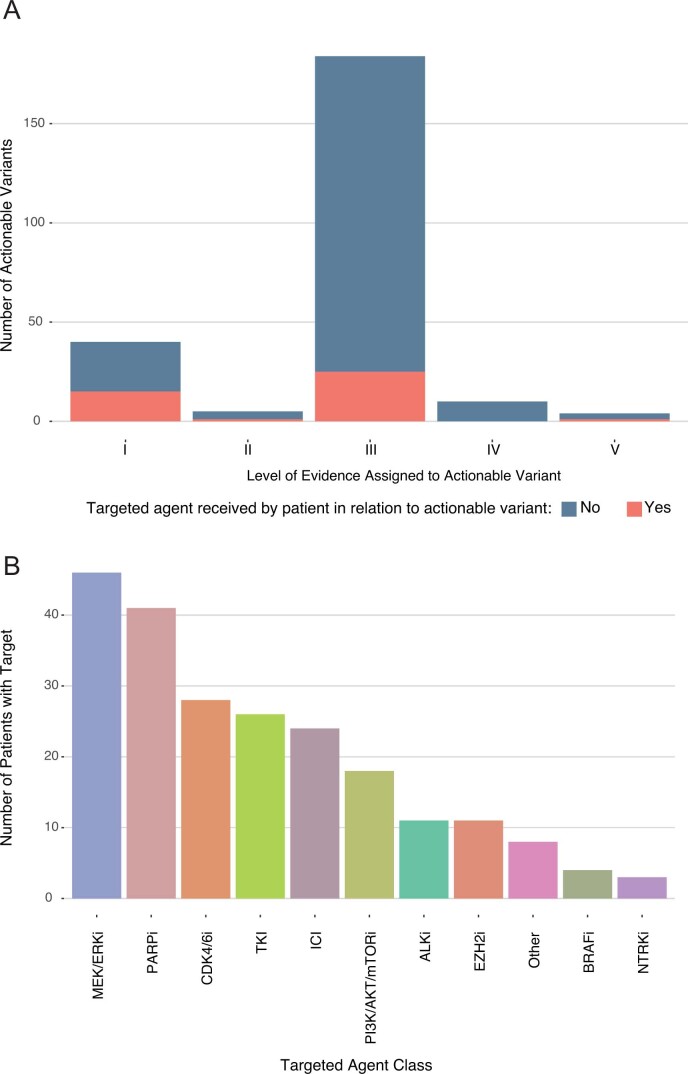
Therapeutic biomarkers in the KiCS cohort. (**a)** Level of evidence for category C variants (therapeutic biomarkers) and proportion targeted with a therapeutic agent (N = 222 variants). Details about levels of evidence are available in Extended Data Fig. 4. (**b)** Frequency of targets in various therapeutic agent classes. Targets in each category (not inclusive list): PARP inhibitors: *BRCA1, BRCA2, PTEN, CHEK2, PALB2, ARID1A, RAD51C, RAD51D, BARD1, BRIP1, ATM, ATRX* variants. MEK/ERK inhibitors: *NF1/2, N/K/HRAS, MAP2K1, PTPN11* variants, *BRAF* fusions. Immune checkpoint inhibitors: Tumor mutational burden >5 mutations/Mb, or biallelic loss of a MMR gene. CDK4/6 inhibitor: *CDK4/6* amplification, *CCND2* or *CCND3* amplification, *CDKN2A/B* homozygous deletion. Growth TKI: *KIT, FGFR, PDGFR, RET, EGFR, VEGFR, FLT3* variants/amplification and *ABL* fusions. ALK inhibitors: *ALK* hotspots, fusions, *MET* and *ROS1* fusions. PI3K/AKT/mTOR inhibitors: *PIK3CA, PIK3R1, AKT1, FBXW7*, *PTEN, TSC1/2* variants, *ARID1A* variants (in addition to genes targetable by MEK/ERK inhibitors above). EZH2 inhibitor: *EZH2, SMARCB1, SMARCA4, ARID1A* variants. BRAF inhibitor: *BRAF* variants. NTRK inhibitors: *NTRK* fusions. Other: IDH1 inhibitor, WEE1 inhibitor, Wnt pathway inhibitor, Hif inhibitor, BRD and BET inhibitor, SHH inhibitor. Source data

**Extended Data Fig. 4:**
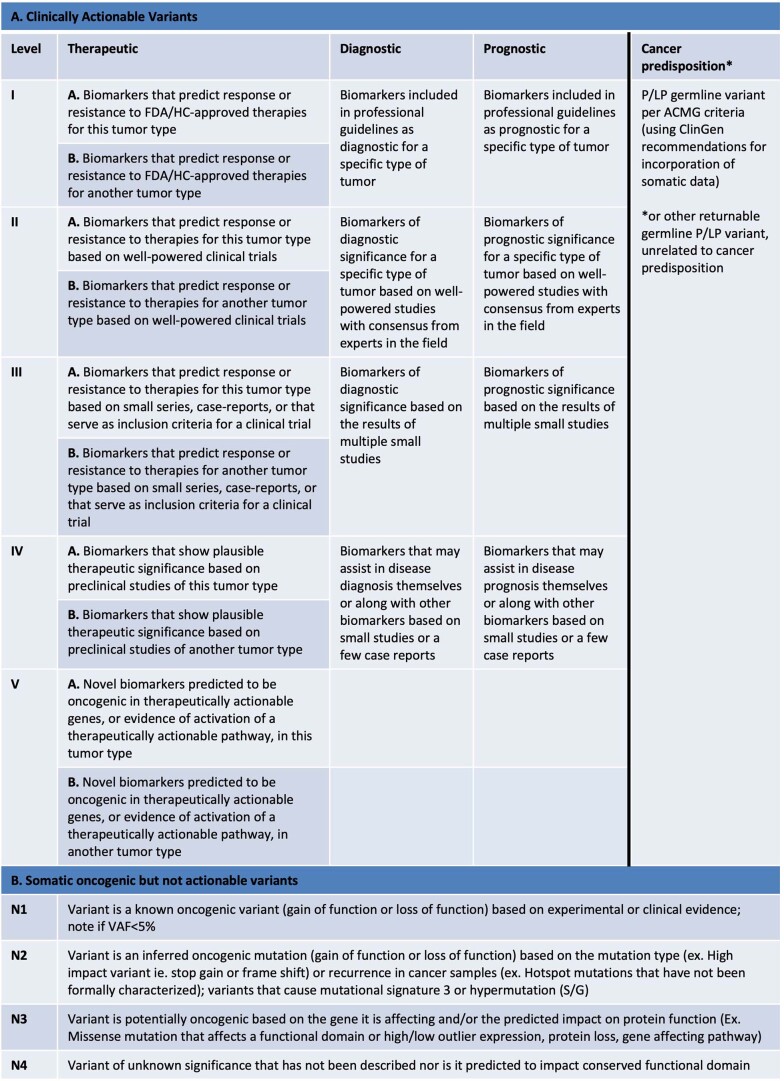
Classification of actionable variants. Classification is adapted from AMP/ASCO/CAP, OncoKB guidelines and NCI/COG Pediatric Match levels of evidence, and with reference to ACMG/ClinGen criteria^62,63,66,67^.

Fifty-four percent of participants with tumor analysis (*n* = 143/264) had sequencing findings that were therapeutically targetable (category C; Figs. 2 and 3a and Supplementary Table 5); within subgroups, this corresponded to 72%, 58% and 46% of patients with a CNS tumor, solid tumor or leukemia/lymphoma, respectively. Fifty-one of the 143 (36%) cases had more than one targetable finding, which were also unequally distributed among tumor classes: 25 of 41 (61%) CNS tumor cases had more than one targetable finding, compared to 8 of 28 (29%) leukemia/lymphoma cases and 18 of 74 (24%) solid tumor cases. Targets of MEK–ERK inhibitors, PARP inhibitors, immune checkpoint inhibitors and cell cycle inhibitors predominated (Extended Data Fig. 3b). Thirty-three participants (23%) had targetable variants derived from the germline. After excluding patients who were on another line of therapy (including targeted agents) or who had stable disease or no evidence of disease when sequencing results became available and actionable, we identified 69 of 143 (48%) patients who were in need of a therapeutic option. Follow-up information was missing for seven patients. Twenty-five patients (25/62, 40%) were not treated with a matched agent for the following reasons: no trial or agent accessible (*n* = 9); end of life (*n* = 15); and patient/family refusal (*n* = 1). Thirty-seven (37/62, 60%) participants were treated with a matched targeted agent, 14 of 37 as part of a clinical trial (including one patient through a single-patient study), 15 of 37 through compassionate access, 7 of 37 through commercial access and 1 of 37 though an unknown mechanism (Supplementary Table 5). While one must acknowledge the potential for selection bias, this represents an impressive proportion of patients who were able to obtain a novel agent on the basis of their sequencing findings. Extended Data Fig. 3a shows the number of actionable variants assigned to each level of evidence (Extended Data Fig. 4) after review by the molecular tumor board, along with the proportion of variants for which a targeted drug was administered. As expected, this proportion was the highest for variants with level of evidence 1 (biomarkers that predict response or resistance to Food and Drug Administration/Health Canada-approved therapies). A majority of variants were attributed level of evidence 3 (biomarkers that predict response to therapies on the basis of small series or case reports or that serve as inclusion criteria for a clinical trial).

Our sequencing approach explicitly incorporated germline analysis given the important implications of germline findings for patients and their families, including the potential for finding drivers with therapeutic relevance for an active cancer^13^. Previously unknown germline likely pathogenic or pathogenic (LP/P) variants in cancer predisposition genes (CPGs) were found in 15% (*n* = 46/300) of participants, resulting in referral for genetic counseling in 89% of cases and cascade testing and initiation of tumor surveillance for participants and/or their relatives in at least 67% and 37% of cases, respectively (9 participants had unknown surveillance status; Supplementary Table 6). In addition, findings for all patients previously identified as having germline LP/P variants in CPGs before referral to KiCS (*n* = 16) were corroborated by our analysis. The majority of new findings were in genes not typically associated with pediatric cancers, including variants in HR pathway genes and heterozygous variants in DNA mismatch repair (MMR) genes. When excluding patients enrolled specifically to investigate a high suspicion for cancer predisposition (EP2) and those with a previously known cancer predisposition syndrome, 17% (*n* = 41/241) of participants had a P/LP germline variant in a CPG, of whom 4 had been previously referred for clinical genetic testing while only 11 had a noteworthy personal or family history of cancer. Among the 43 participants specifically enrolled for suspicion of a cancer predisposition syndrome (EP2), only 5 (12%) had new findings on the cancer panel.

**Fig. 4:**
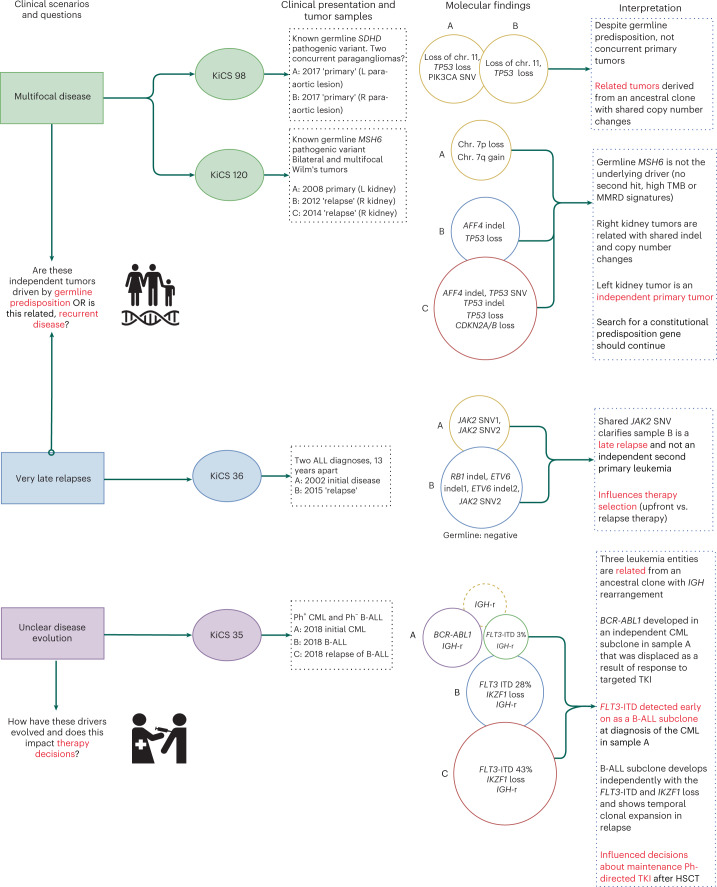
Comprehensive sequential tumor analysis provides important insights into the relationship between multiple neoplasms of the same histology presenting in the same individual (category Dii). Results are shown for *n* = 4 patients. HSCT, hematopoietic stem cell transplantation; *IGH*-r, *IGH* rearrangement.

Comprehensive sequential tumor analysis also provided important insights into the relationship between multiple neoplasms of the same histology presenting in the same individual (*n* = 4) (category Dii; Fig. 4). For instance, in one patient, a second episode of acute lymphoblastic leukemia (ALL) from 13 years after the original occurrence was determined to be a very late relapse rather than a new leukemia on the basis of a shared *JAK2* driver variant and was therefore treated with relapse therapy. In another case, a unique clonal evolution trajectory was discovered in a patient with chronic myeloid leukemia (CML) and two subsequent events of B cell ALL (B-ALL). These three leukemia entities were derived from an ancestral clone with *IGH* rearrangement, rather than *BCR-ABL1* fusion, which developed in an independent CML subclone. A *FLT3* internal tandem duplication (ITD) and *IKZF1* deletion developed in an independent B-ALL subclone and showed temporal clonal expansion in relapse. *FLT3*-ITD was detected early on in the diagnostic CML sample at a subclonal variant allele fraction (VAF) of 3%. These findings explained the unusual lack of *BCR-ABL1* fusion in the B-ALL malignancies and also influenced decisions regarding maintenance imatinib after hematopoietic stem cell transplantation. By identifying common clonal variants or the evolution of subclonal variants, it was possible to distinguish late relapses from independent primary lesions and to map divergent neoplastic processes from ancestral clones. Elucidating such biological underpinnings has very practical clinical impacts, influencing choice of therapy, qualifying concerns for cancer predisposition (which would be heightened in the setting of independent primary tumors) and potentially surveilling for expansion of minor subclones.

### Somatic WGS detects additional clinically relevant findings

Seven additional clinically actionable variants were identified upon retrospective analysis of somatic WGS data that were not detected by cancer panel or RNA-seq analysis (Supplementary Table 7). ITDs involving exon 15 of the *BCOR* gene in a CNS neuroepithelioma and a renal tumor suggested revised diagnoses of high-grade neuroepithelial tumor with *BCOR* alteration and clear cell sarcoma of the kidney, respectively. Five therapeutic targets were identified, including in *CDK4* (intragenic duplication involving the kinase domain) with corroborating outlier gene overexpression and in *PTEN* (translocation/inversion event and promoter swap), *NF1* and *SMARCB1* (complex structural variants) with outlier underexpression. This WGS analysis was also fruitful (Supplementary Table 7) in detecting second hits in tumor-suppressor genes, demonstrating interesting mechanisms of tumor-suppressor inactivation and complex fusion events and highlighting a number of novel variants. In the absence of functional data, however, the clinical actionability of such novel variants is limited.

### Disturbed DNA repair pathways drive pediatric cancer onset and evolution

In adult patients with cancer, *BRCA1* and *BRCA2* variants are tissue-restricted biomarkers for PARP inhibitor sensitivity^12^. Although germline variants in *BRCA1*, *BRCA2* and other HR repair (HRR) pathway genes have been described in large sequencing studies of pediatric oncology patients^7,14^, the relevance of these findings to pediatric cancer pathogenesis remains unclear. In our cohort, nearly half of the germline LP/P variants in CPGs (21/46) occurred in genes involved in HRR (*BRCA1*, *BRCA2*, *PALB2*, *BARD1*, *CHEK2*, *ATM*, *BLM*, *RAD51C*, *FANCA*, *FANCC* or *ERCC4*; Supplementary Table 6). To clarify the contribution of these variants to tumor pathogenesis in these individuals, we conducted somatic signature analysis of 293 tumor samples with available WGS data in the KiCS cohort. The proportion of Catalogue of Somatic Mutations in Cancer (COSMIC) single-substitution signature 3 (SBS3; BRCAness mutational signature; requirement for a minimum of 100 mutations contributing to this signature) in KiCS was compared to that in a cohort of sporadic cancers for which WGS was performed (Pan-Cancer Analysis of Whole Genomes (PCAWG))^15^. Signature 3 was detected in 13.3% (369/2,780 samples) of the mostly adult samples in the PCAWG data, which is lower than the 23.1% (56/242; *P* = 0.00006) of ‘HR-negative’ KiCS samples (those without somatic or germline mutations in HRR genes) that exhibited this signature. However, the prevalence of signature 3 was significantly higher among KiCS samples with somatic mutations affecting the HR pathway (‘KiCS HR somatic’; 8/26 samples or 30.8%; *P* = 0.01) and highest in KiCS tumor samples from patients with germline LP/P variants affecting the HR pathway (‘KiCS HR germline’; 12/25 samples or 48%; *P* = 0.00003; Fig. 3b). Detailed analyses of KiCS HR somatic and KiCS HR germline cases are presented in Supplementary Table 8. Loss of heterozygosity (LOH) of the germline variant was found in a minority of pediatric tumors and did not always correlate with the presence of signature 3. Many of these tumors, however, showed somatic copy number loss in other components of the HR pathway. The effect of these additional losses on HR pathway function is unclear. To further interrogate our findings of HR deficiency (HRD), we used the HRDetect algorithm^16,17^ to calculate HRD probability scores for KiCS samples with WGS data (*n* = 290). Sixty-eight tumors (23%) had an HRD probability score higher than 70% (the probabilistic cutoff suggested to predict *BRCA1* and/or *BRCA2* deficiency)^17^. While both approaches indicated HRD in a sizable number of cases, they did not always agree: we found no meaningful correlation between the samples with a high HRD score and those we previously determined to harbor SBS3.

**Fig. 5:**
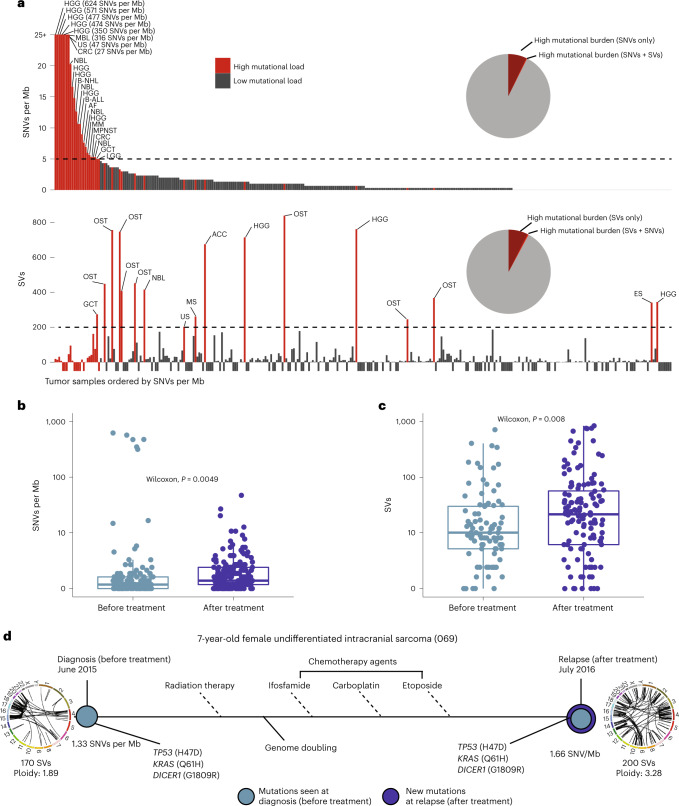
Impact of therapy on TMB in pediatric and AYA cancers. **a**, Bar charts: KiCS tumor samples ordered by SNVs per Mb, displaying SNVs per Mb and structural variant (SV) count on the *y* axis. Red bars indicate samples that had a high mutational load for at least one of the two mutation types. Pie charts: combined red slices indicate the proportion of samples with high mutational load for respective mutation type. The dark red slices indicate the proportion of samples with a high mutational load for only one of the mutation types (that is, not high for the other mutation type). For **a**, *n* = 326 individual tumor samples from 249 patients. **b**,**c**, Box plots showing SNVs per Mb (**b**) and structural variant count (**c**) for samples obtained before treatment versus after treatment (chemotherapy and/or radiation). Wilcoxon two-tailed *P* values are shown. Box plots show quartiles with the center line representing the median and whiskers representing 1.5 times the interquartile range. For **b**, *n* = 326 individual tumor samples from 249 patients; test statistic (*z* score) = 2.81; effect size = −0.156. For **c**, *n* = 217 individual tumor samples from 180 patients; test statistic (*z* score) = 2.65; effect size = −0.180. **d**, Example of a tumor sampled before and after treatment. Light blue indicates mutations seen before treatment and dark blue indicates new mutations present at relapse (after treatment). Source data

As altered DNA repair pathways can result in accumulation of tumor mutations, we proceeded to evaluate somatic tumor mutational burden (TMB) in 249 patients. Nine percent of participants (*n* = 22 with 23 tumors) had hypermutant tumors, 9 with a TMB of 5–9.9 mutations per Mb, 7 with a TMB of 10–99.9 mutations per Mb and 6 with ultra-hypermutation (TMB of >100 mutations per Mb; Supplementary Table 9). An additional 8% of participants (*n* = 14 of 179 participants with structural variant counts) exhibited high levels of structural variants (>200) (Fig. 5a). Only 36% (*n* = 8) of patients with hypermutant tumors had LP/P germline variants in MMR genes (*n* = 3 heterozygous, *n* = 1 compound heterozygous, *n* = 4 homozygous; Supplementary Table 6), and one patient had somatic biallelic deleterious variants in MMR genes. In 45% (*n* = 10) of patients, MMR deficiency was not documented; instead, these patients had a substantial burden of prior therapy (second primary tumors (*n* = 4) or relapsed/multiply progressed tumors (*n* = 6)). Accordingly, samples acquired after therapy showed significantly elevated mutational burden compared to pretherapy samples (Fig. 5b,c).

### Clonal evolution at relapse provides new therapeutic options

**Fig. 6:**
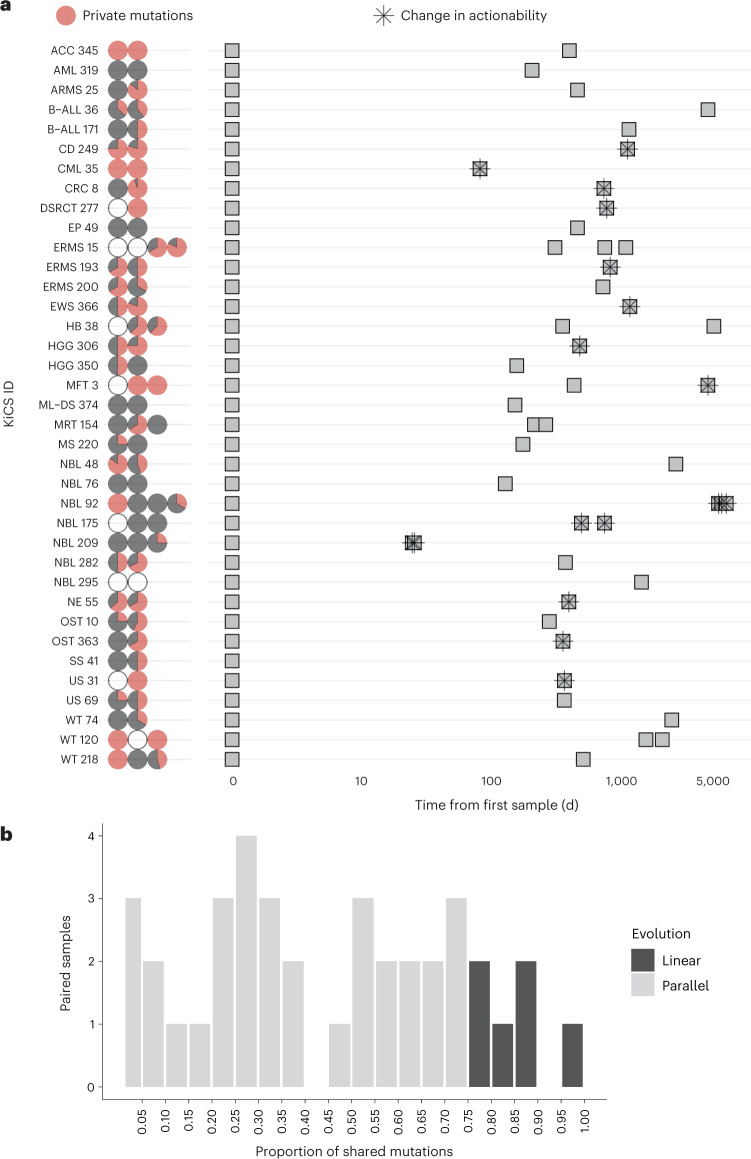
Evolution of childhood cancers across time. **a**, Each row corresponds to a patient with a single tumor diagnosis (*n* = 38 patients). Pie charts represent samples analyzed by cancer panel for each tumor where SNVs were detected at a VAF of greater than 0.10. Pie charts are colored by the proportion of mutations identified at each time point that were private to that sample (red) or shared with at least one other sample for that tumor, at a VAF of greater than 0.05 (black). Open circles represent no SNVs detected above the threshold. The center panel depicts samples (gray squares) in sequential order with time on the *x* axis, showing the number of days since the initial sample was obtained. A star represents the emergence or loss of a targetable driver, leading to a potential change in clinical action. Note that samples obtained at the same time point (days since diagnosis) correspond to anatomically distinct lesions (for example, local relapse versus lung metastasis). **b**, Proportion of mutations shared by each primary tumor with its paired relapse (*n* = 25 individual patients with 38 initial tumor–relapse pairs). Using WGS data, initial tumor samples were compared to relapse samples (with a one-to-one comparison comprising a ‘pair’). The proportion of SNVs from the initial sample shared with the paired relapse sample is characterized as parallel or linear on the basis of a 75% threshold^29^. Source data

**Extended Data Fig. 5:**
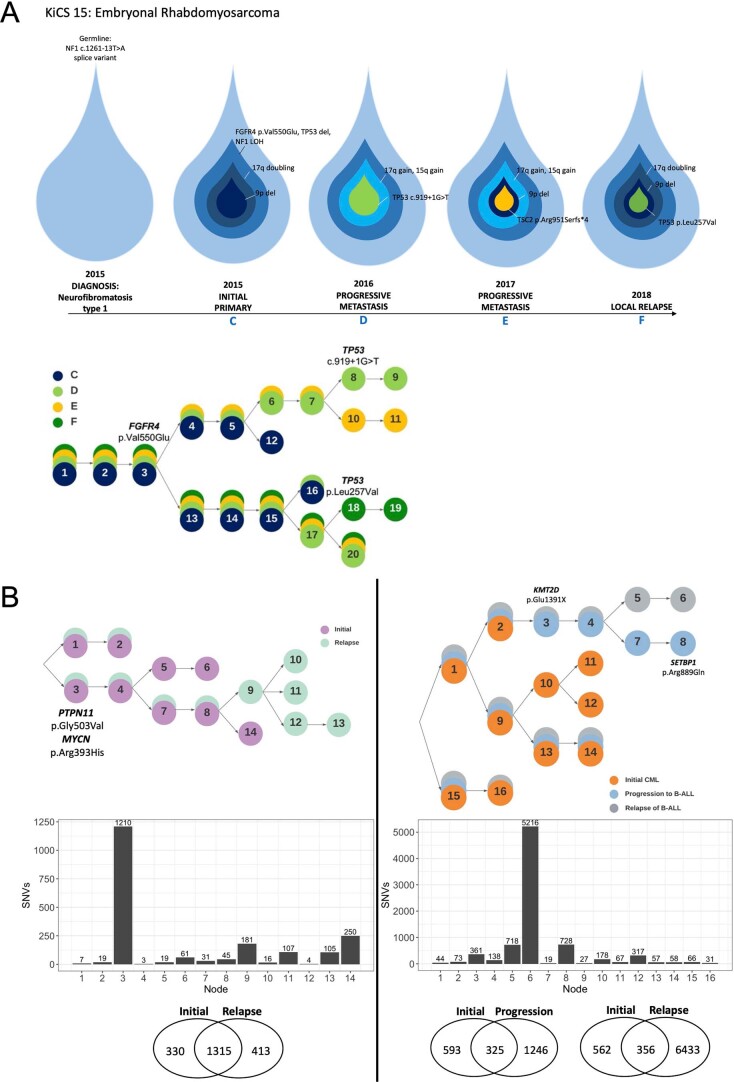
Examples of clonal evolution in the KiCS cohort. **(a)** Clonal evolution of an embryonal rhabdomyosarcoma in an individual with constitutional Neurofibromatosis-1 (KiCS 15). Upper panel: Ancestral ‘drivers’ are detected early and maintained through tumor evolution regardless of spatial evolution (*FGFR4* SNV, chr17p-). New clones emerge at progression with additional SNV/CN changes. Tumor progression within the same spatial region show significant SNV/CN commonalities (D and E), while others despite temporal/spatial changes show important similarities with ancestral clones (C and F). Lower panel: The phylogenetic tree annotated with SNV drivers. Each node represents a subpopulation whereby arrows extend from ancestors to descendants. This is supportive of the findings that although ancestral driver events may diminish or be displaced during tumor progression, they may also be maintained as subclones that drive relapse. (**b**) Parallel and linear evolution in multi-sample KiCS cases (N = 2 individual cases; one case shown on each side of the vertical line). Phylogenetic trees from WGS annotated with SNV drivers, number of SNVs comprising each tree node, and the number of SNVs unique or shared between paired initial and subsequent tumor samples. Left panel: KiCS 319 represents linear evolution, with 79.9% of initial SNVs shared with the relapse. In the KiCS cohort, linear evolution is uncommon; only 4 patients shared >75% SNVs between initial and relapse tumor samples. Right panel: Chronic myelogenous leukemia (CML) was initially diagnosed in January 2014 for KiCS 35, followed by development of precursor B-ALL sampled in April 2014 and a relapse of B-ALL in July 2018. The initial CML shares 35.4% and 38.8% of SNVs respectively with each subsequent sample. KiCS 35 displays extreme branching and parallel evolution.

The contributions of therapeutic exposures and tumor evolution together may culminate in the emergence or diminishment of tumor drivers at progression/relapse or in metastatic sites^18–22^. In the context of a precision medicine program, further clarification of the frequency of molecular changes during evolution of the tumor is critical. This information can inform whether rebiopsy at relapse or of metastatic sites is warranted as it offers the potential to identify new therapeutic targets that were not present in the primary tumor. To investigate this, we compared DNA cancer panel data from the tumors of 38 patients (*n* = 25 solid tumors, *n* = 6 CNS tumors, *n* = 7 leukemias and lymphomas) for whom multiple sequential samples were available. Notably, we found that the tumor genomes showed substantial changes over the disease course, with the majority of mutations identified at only one sample time point (Fig. 6a). Additional clonality analysis showed a predominance of parallel (versus linear) evolution (Fig. 6b and Extended Data Fig. 5). Through detailed analysis of single-nucleotide variants (SNVs), insertions/deletions (indels) and copy number changes, we determined the proportion of patients with changes in tumor drivers (new drivers, expansion of driver clones, displacement/loss of driver clones or diminished driver clones; Supplementary Table 10). A detailed analysis of one patient with recurrent rhabdomyosarcoma is presented in Extended Data Fig. 5a. Of 38 patients, 22 showed emergence of a new driver and 9 showed loss of a driver; when considering the subset of these with therapeutic implications, 37% of patients (*n* = 14) had a change in therapy recommendation as a result of serial sample analysis (Fig. 6a and Supplementary Table 10).

## Discussion

In this precision oncology study of pediatric and AYA patients, we present sequencing and comprehensive clinical annotation of a wide array of CNS tumors, solid tumors and hematological malignancies. Our analysis extends to multiple samples per participant, many of which are from after initiation of therapy and from sites of progressive, recurrent or metastatic disease. We demonstrate clinical actionability beyond the identification of therapeutic targets and provide insights into the contribution of germline drivers and cancer therapies to the pathogenesis and evolution of pediatric cancer. We also highlight the utility of rebiopsy at relapse, on the basis of evidence of changes in therapeutically targetable drivers.

Collectively, this study demonstrates both the feasibility and utility of incorporating comprehensive somatic–germline genomic analysis into pediatric oncology care. Uptake of testing was high (95% consent rate), and sufficient tissue was available for analysis in 94% of patients. The clinical actionability of the findings was broad: although the identification of novel therapeutic targets in patients with hard-to-cure cancers remains a primary indication for genomic analysis (found in 54% of patients in this cohort; Fig. 2b), we demonstrate that comprehensive sequencing also improves upfront diagnostics and prognostication. Our approach was instrumental in refining or changing the diagnosis for 17 uncertain cases in this cohort (Table 1), which permitted the appropriate choice of antineoplastic therapy, rather than exposing patients to ineffective therapies and their side effects. Notably, our analysis also reliably captured every canonical fusion and other clinically relevant markers (for example, *MYCN* amplification in neuroblastoma), while providing further detailed characterization of clinical findings in other cases (Supplementary Table 3a,b). NGS-based tumor analysis can provide more comprehensive assessments than standard clinical molecular/cytogenetic testing and could eliminate the time delays^23^ inherent to performing sequential assays (Table 1), particularly for cases with diagnostic uncertainty or for which tumor tissue is limited (small biopsies from pediatric patients). Notably, our approach is flexible and adaptable in accommodating new diagnostic and prognostic markers that may be prioritized for identification and risk stratification in the future, without the need to develop and validate new assays.

Our prospective analysis was based on a comprehensive cancer panel and full transcriptome sequencing. Retrospective analysis of somatic WGS data in our cohort, using a filtering strategy designed to detect variants that would be overlooked by a panel, identified a small number of additional clinically actionable findings (*n* = 7; Supplementary Table 7). A combined sequencing approach that includes WGS has been shown to be most sensitive by other groups^24,25^ and permits comprehensive integrative analysis (including of mutational signatures). This is likely to be a preferred approach moving forward, depending on the goals of the specific sequencing effort, technical ability and financial constraints. Not surprisingly, WGS analysis also led to identification of other biological drivers, improved mechanistic insight into complex structural variation and detection of novel variants in regulatory and noncoding areas, which will inform future discovery and potentially future clinical care.

Two important observations emerge from our analysis that challenge current tenets of pediatric cancer biology. The first is the presence of a notable proportion of high mutation burden in relapsed childhood cancers, across all classes of mutation (Fig. 5). This is in distinct contrast to the ‘quiet’ pediatric genomes described in prior studies^2,26^, which are largely based on assessments of diagnostic tumor specimens, and thus represents an important addition to the literature. This finding is not exclusively associated with germline pathogenic variants in genes associated with MMR deficiency. We demonstrate that tumors exposed to chemotherapy and radiation have a significantly higher mutational burden than pretherapy samples (Fig. 5b,c), which has been shown in prior studies of paired primary–relapse tumors^22^ of specific histologies, including neuroblastoma^27,28^ and glioma^29^, and is now observed across a broad range of tumor histiotypes in our cohort. These findings suggest a potential role for immune checkpoint inhibitors in subsets of pediatric tumors, as hypermutation is evolving as a biomarker of immune checkpoint inhibitor efficacy, including non-SNV neoantigens^30–34^. In recent work, response to immune checkpoint inhibitors was associated with TMB across a wide range of cancer types, particularly among those with TMB in the top 20% for a given histology^30^. Accordingly, TMB of more than 5 mutations per Mb may be a more appropriate cutoff to define hypermutation in pediatric tumors (and TMB as low as 2 mutations per Mb has been suggested to represent ‘pediatric highly mutated’ in diagnostic cohorts^2^), given the low SNV burden among tumors from the overwhelming majority of these patients; we are actively pursuing this question in a clinical trial^35^. We also conducted a detailed analysis of changes to molecular drivers at the time of relapse (Fig. 6 and Supplementary Table 10), which extends findings described in histology-specific cohorts^18,27,28,36,37^ and patient-derived xenograft models^38^ and so far has been shown in only very small patient subsets of a few broad precision medicine studies^22,39–43^, with limited analysis. Collectively, these data lend support for repeat profiling of relapsed pediatric cancers after therapy^44^, which may identify new drivers or genomic architecture to target. Future analysis, including assessment of therapy-related mutational signatures, will help clarify the extent to which increased mutational load and detection of new drivers in the relapse setting is due to therapy-induced pressures.

The second notable finding in pediatric cancers is the prevalence of defects in the HRR pathway, suggesting potential utility for PARP inhibitors (Extended Data Fig. 3b). Indeed, deficient DNA repair has been identified to be an important contributor to pediatric cancer pathophysiology and has been explored in a number of specific cancer types, including in neuroblastoma and osteosarcoma^45,46^. In this study, we focused our analysis on tumors with identifiable driver variants in the HRR pathway. Several variants originate in the germline, and their contribution to pediatric cancer pathogenesis has, thus far, remained unclear^3,7,14^. Early epidemiological studies highlighted an association between women with breast cancer and sarcomas in their relatives, and a high prevalence of childhood cancers in families with mutations in *BRCA2* has also been described^47–49^. The presence of pathogenic variants in HRR genes in the germline of pediatric patients with cancer has been reported in specific tumors^50–52^, in germline studies of broad populations of pediatric oncology patients^7,8,14,53,54^ and in some precision medicine studies^3,6,25,39,42^. We present an in-depth correlative analysis to interrogate the contribution of such germline variants to pediatric cancer pathogenesis. Herein we demonstrate an enrichment in mutational signature 3 in the tumors of patients with germline LP/P variants in HRR genes (Fig. 3b), providing support for their role as drivers of pediatric cancers, despite the apparent lack of a second somatic hit in many. It is possible that other mechanisms of second allele inactivation are at play, including epigenetic silencing, dominant-negative effects or post-translational modifications. Direct application of the HRDetect score did not yield robust correlation with mutational signature analysis and/or inactivating mutations in HRR genes. While the interpretation of signature 3 requires a cautious approach, we also strongly feel that this finding should not be dismissed. Collectively, these findings will continue to motivate the pediatric oncology community to investigate further and determine more reliable assessments of HRD in pediatric cancers and interpretations of existing measures.

Analysis of the pediatric germline is a critical component of a comprehensive sequencing effort. As noted above, a number of potentially important therapeutic targets were identified from the pediatric germline (Fig. 3a). Furthermore, identification of germline mutations in CPGs was not strongly correlated with a personal or family history of cancer^7^, and cancer predisposition would have remained undiagnosed if the germline had not been independently sequenced. The impact of these findings often extends beyond the pediatric patients themselves and stands to benefit parents and family members through the institution of appropriate tumor surveillance measures^55,56^ where indicated and family planning considerations, as was shown in our cohort. The proportion of patients in our study with germline LP/P variants in CPGs (15%, not including already known germline variants) is moderately higher than reported in some other studies but is fairly consistent with others^3–8,14,39,40,53,57–59^; these proportions, which range from 8% to 35%, are influenced by the breadth of the sequencing platforms used and genes reported, ascertainment bias (tumor types, ancestries) and the inclusion or exclusion of carrier status in frequency counts. Of particular interest in this study was the application of broad cancer panel sequencing to the germline DNA of patients with high clinical suspicion for cancer predisposition but with negative targeted testing to date (EP2). A pathogenic variant in a known CPG was reported in 5 of 43 (12%) patients. Although these variants may represent the causative drivers of the suspicious personal or family cancer history in these five patients, these findings suggest that other mechanisms such as yet-to-be-discovered genes, variants that span regulatory regions, epigenetic changes or polygenic interactions from multiple low-penetrant variants in a common pathway should be considered to explain cancer predisposition risk more generally.

In summary, the application of comprehensive somatic–germline analysis to pediatric oncology care conveys a breadth of clinical utility, extending beyond identifying canonical targets, which provides a rationale and urgency for its incorporation into standard clinical practice for all pediatric and AYA patients, at diagnosis and at relapse^24^. Given the relatively small numbers of these patients, population-wide sequencing for every child and young adult with cancer is likely to be a realistic goal. While stratified approaches may be considered (for example, transcriptome analysis at diagnosis for solid tumors and leukemias), it is clear that integrative, comprehensive tumor–germline sequencing provides the broadest impact (Fig. 2b). This applies particularly to the interrogation of therapeutic targets, which show no consistent association across most tumor types (Fig. 3a). Challenges remain in performing NGS for specific patient populations (for example, tumors with poor cellularity such as in Hodgkin lymphoma and patients with relapsed hematological malignancies after allogeneic transplantation). Finally, we demonstrate how a program that adds careful clinical annotation to sequencing efforts can also rapidly accelerate meaningful biological insights. Comprehensive approaches such as these along with data sharing efforts will advance the understanding of pediatric and AYA cancer pathogenesis and evolution and improve patient management and outcomes.

## Methods

This study was approved by The Hospital for Sick Children Research Ethics Board.

### Patient recruitment

Launched in April 2016, KiCS is an ongoing prospective study. We report on the first 300 participants analyzed. KiCS is available to patients at The Hospital for Sick Children but also enrolls nationally and internationally. Patients are referred by their primary oncology teams to one of two streams: EP1 (tumor + germline analysis), including individuals with a difficult-to-cure cancer (any metastatic, poor prognosis (predicted 5-year overall survival <50%) or relapsed tumor) or a poorly characterized rare tumor and patients for whom NGS could answer a clinically relevant question that had not been addressed by clinical testing, and EP2 (germline ± tumor analysis), including individuals suspected for a cancer susceptibility syndrome on the basis of personal or family history, with negative targeted clinical testing. All participants (or substitute decision-makers) provided informed consent. Participants received no compensation. The sex and age of participants included in various analyses can be determined by referring to Supplementary Tables 1 and 2.

### KiCS program overview

Paired tumor–normal sequencing was conducted. Fresh frozen tumors were required initially, but an amendment to accept FFPE samples was incorporated in May 2018. Normal (‘germline’) samples were derived from peripheral blood. For individuals with hematological malignancies, DNA was extracted from skin biopsy-derived short-term cultured fibroblasts or a peripheral blood sample if negativity for minimal residual disease was confirmed in the bone marrow. Otherwise, we used maternal and paternal blood. In brief, tumors were analyzed against each parent’s germline independently. Variants common to both analyses were considered true somatic tumor variants. Germline variants that were maternally or paternally inherited, found in only a single analysis, were excluded. It was not possible to ascertain germline de novo variants in these patients. When possible, the germline origin of variants was assumed on the basis of allele frequency or presence in population databases (gnomAD); however, variants were mostly reported as somatic findings. If available, the percentage of malignant cells in the tumor sample was noted to further filter out variants from the somatic analysis. Particular consideration was given to the analysis of relapsed leukemia in patients after allogeneic bone marrow transplantation^60^. Preanalytic removal of donor cells from mixed tumor–donor specimens (for example, by flow cytometry) was considered but not always feasible. If available, donor DNA derived from the patient (*f*or example, a remission blood sample taken after transplantation) or donor blood (if available for consent (for example, from a sibling donor) was sequenced to filter out variants from the somatic analysis. In the absence of these, the percentage of donor chimerism was noted for analysis purposes.

Tumors were sequenced using a custom pan-cancer DNA panel providing deep coverage for 864 cancer-associated genes (Supplementary Table 11) and with whole-transcriptome (RNA-seq) sequencing and WGS. Paired germline samples were analyzed by panel (with specific use of a CPG list for reporting; Supplementary Table 12) and WGS. For individuals in EP2, tumor specimens were analyzed when possible to complement germline analysis. Sample preparation and panel sequencing were performed in a CAP/CLIA-accredited laboratory.

All sequencing data were annotated with comprehensive clinical details, abstracted from medical records, chemotherapy charts/protocols and institutional clinical databases. ICD morphology codes (ICD-O-3.1)^61^, when not provided on the pathology report, were assigned retrospectively by two pediatric oncologists. Data were collected on any systemic (intravenous/oral) cancer-directed therapies received more than 24 hours before resection of the analyzed tumor specimens. Therapeutic radiation exposures were captured, and two study coinvestigators categorized each tumor specimen as ‘in field’ or ‘not in field’ of prior radiation.

**Extended Data Fig. 6:**
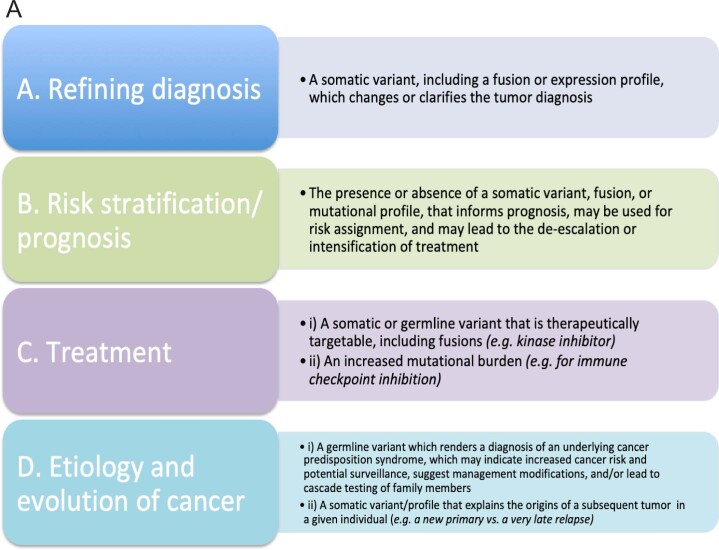
Categories of clinical actionability used to broadly classify sequencing variants. Four classes of actionability were considered to classify the clinical relevance of sequencing variants.

Findings from the panel and RNA-seq were discussed at a weekly multidisciplinary molecular tumor board, whose members included genome scientists, bioinformaticians, oncologists (with hematological, neuro-oncology, solid tumor and experimental therapeutics expertise), clinical geneticists, genetic counselors, laboratory geneticists and pathologists. The clinical actionability of sequencing data was categorized as shown in Extended Data Fig. 6. Each actionable variant was individually counted. Variants with more than one aspect of actionability were counted once but were recorded in each relevant subcategory for clarity. Actionable variants were categorized using a classification framework, based on AMP/ASCO/CAP and OncoKB guidelines and NCI-COG Pediatric Match Levels of Evidence (Extended Data Fig. 4), that was adapted to include signatures and hypermutation^62,63^. These data were recorded in REDCap (v11.0.5). Clinically validated actionable findings were returned to the referring clinician for disclosure.

WGS data were used to corroborate TMB and to assess mutational signatures^64^. Somatic WGS data were further interrogated retrospectively, using a strategic filtering approach to identify variants that would be missed by the panel, including small deletions/duplications, variants overlapping upstream/regulatory or noncoding regions of a gene and complex structural variants. Variants already detected by the cancer panel and/or RNA-seq were removed, and additional clinically actionable findings were recorded.

### Cancer panel DNA extraction, library preparation and sequencing

DNA from 1.0-ml EDTA blood vacutainer tubes was extracted (DSP Blood Midi Kit with QIA Symphony DNA extractor, Qiagen). A custom 1.0-ml EDTA blood protocol was run on the QIA Symphony with DNA eluted in 200 μl TE (Qiagen, without azide). DNA from bone marrow aspirates, fresh and frozen tumor tissues, and blood (volume of <1.5 ml or with low white blood cell count) was extracted with the QIAamp Micro silica-based membrane column kit (Qiagen). Specimen input of 1–8 mg of tumor tissue, 50 μl of bone marrow aspirate or 100 μl of blood was extracted on a single QIAamp microcolumn and eluted in 20 μl TE. DNA was extracted from FFPE tissue using the QIAamp DNA FFPE tissue kit (FFPE tumors were included for participants 92, 170, 200, 209, 228 (2 samples), 269, 273 (2 samples), 299, 306, 358, 362, 370, 375, 376, 377, 384 and 400). DNA was quantitated with a Biomek FLX800 fluorimeter using 2 μl DNA, diluted in 198 μl assay B solution containing Hoescht dye (Sigma). A standard curve of 25–450 ng DNA was prepared using commercial genomic DNA (Sigma), and patient DNA was quantified using this curve. Using the SureSelectXT kit, samples were processed using 200 ng DNA. After shearing the DNA (Covaris sonicator), Illumina HiSeq 2500-compatible libraries were generated. Samples were pooled in multiples of six.

### Design and validation of a pan-cancer sequencing panel

The panel was designed to capture more than 15,000 exons from 864 genes (Supplementary Table 11) using Agilent SureSelect. Probes were designed to capture exons and a substantial portion of intronic sequence. Probes with poor coverage were redesigned, especially if they fell within childhood cancer-associated genes. The curated list of associated genes included 514 from COSMIC (v69), 319 from other cancer-specific databases and resources, and 45 from pediatric-specific sequencing manuscripts. We designed probes for the full coding regions of each gene and the promoter of *TERT*, as well as a small number of microsatellites. We compared each probe to those already designed as part of Agilent’s exome kit and ‘boosted’ those with poor coverage in the exome. Some targets were excluded because they consistently performed poorly (>50% of 137 samples with coverage of less than 50× for <95% of target bases) or were duplicated. In total, 864 genes performed consistently, with more than 95% of target bases with over 50× coverage.

Clinical validation of germline and somatic variants (substitutions, insertions and deletions) was performed according to CAP (CAP Molecular Pathology Checklist; MOL.36115 rev.201708) and IQMH (IQMH Medical Laboratory Accreditation Requirements; v7.1, 2017) guidelines. Validation of germline variant calling using the panel was performed by testing the accuracy and reproducibility of variant calls using reference and patient samples with known variants (verified by orthogonal clinical methods). Only variants found in genomic intervals common to both methods were used. This was done in triplicate within and between sequencing runs, ensuring ≥95% and ≥85% sensitivity for substitutions and indels, respectively.

Validation of somatic variant calling was performed using patient samples and mock tumor samples, created by mixing reference samples. Samples were mixed to include varying dilutions of the mock tumor in the mock germline–normal sample, yielding somatic variants with a range of VAF values to test variant calling and to challenge the limit of detection of the panel. These variants were also visually curated to derive a gold-standard truth set. Limit of detection was also assessed by attempting to detect variants with low VAF for patient material verified by a clinically validated orthogonal method. The accuracy and reproducibility of variant detection was tested within and between sequencing runs, ensuring ≥90% sensitivity for substitutions and ≥80% sensitivity for indels with the limit of detection challenged and reproducible down to 5% VAF for substitutions and 10% VAF for indels.

### Processing of cancer panel sequencing data

Tumor and non-neoplastic DNA samples were sequenced using the following thresholds: ≥98.5% of bases at ≥50× depth; ≥95% of bases at ≥200× depth; and ≥75% of bases at ≥500× depth (for fresh frozen samples). FASTQ files were aligned to hg19 (BWA-MEM v0.7.15). PCR duplicates were marked with Picard (v2.5.0), with indel realignment and recalibration of base quality scores using GATK (v3.6.0). The mean coverage across the cancer panel achieved with these parameters for the cohort was 1,181× for fresh frozen tumor and blood samples and 829× for FFPE tumor samples. NGSCheckMate^65^ was used to ensure tumor and germline pairs were from the same patient.

### Cancer panel variant detection

Germline substitutions and indels were called using GATK HaplotypeCaller (v3.6.0) with a minimum base quality score of 20 and a minimum confidence threshold of 30. Germline substitutions were further filtered using the following rules: QD < 2.0, FS > 60.0, MQ < 40.0, MQRankSum < −12.5, ReadPosRankSum < −8.0 and SOR > 30. Germline indels were filtered using the following rules: QD < 2.0, FS > 200.0, ReadPosRankSum < −20.0 and SOR > 10.0. Variants were annotated with Annovar and snpEff.

Somatic substitution and indel calling was performed using Mutect (v1.1.4) and Mutect2 (GATK v3.5.0), respectively. Somatic substitutions were excluded using the following rules: variant allele depth in tumor <10, variant allele depth in germline ≥3, reference allele depth in germline ≤50 and VAF in tumor <0.01, VAF ≥0.01 in normal individuals (using multiple control datasets), and germline depth <10 and tumor depth at the position <10, unless the variant passed all Mutect filters. The remaining substitutions were classified as being of high quality if they passed the Mutect internal filters and had a VAF of ≥5%, with at least 50× depth at the position in the tumor and normal samples. Indels were classified as being of high quality if they had a VAF of ≥10%, with >10 alternative reads in the tumor, >50 reference reads in the normal sample and ≤2 alternative reads in the normal sample. Variants from coding sequences and intron–exon boundaries, including 10 bp of intronic sequence for substitutions and 20 bp of intronic sequence for indels, were annotated by Annovar and investigated. The threshold for reporting clinically actionable variants was a VAF of 5% for substitutions and 10% for indels. Exceptions were made for clinically actionable somatic variants detected below these thresholds.

### Reference genome

The clinical cancer panel and research genomes were aligned to hg19.

### Automated variant prioritization using an internal website and database

**Extended Data Fig. 7:**
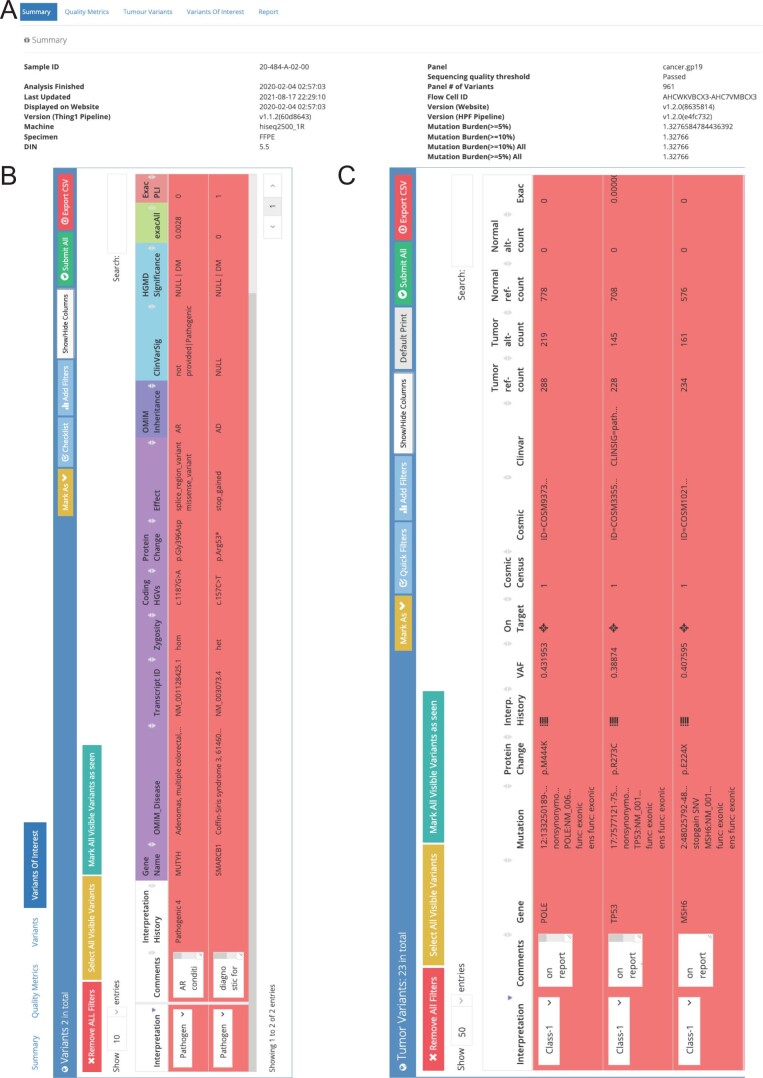
KiCS program internal website and database for automated variant prioritization. **(a)** Summary and Quality Metrics tab on website. (**b)** Germline website – Variants of Interest tab. (**c)** Somatic website – Variants of Interest tab.

A website was developed for variant interpretation, which was used by genome analysts to prioritize germline and somatic variants by applying customizable filters. Summary and quality metrics tabs displayed specimen information, coverage metrics and TMB (Extended Data Fig. 7a). Annotated variants were loaded into a database (MySQL) and displayed on the variants page. Columns for germline variants included mutation type, population frequency, OMIM disease association, mode of inheritance, ClinVar and HGMD classifications, zygosity, read depth and in silico predictions of functional effect (Extended Data Fig. 7b). Additional columns for somatic variants included VAF and COSMIC (Extended Data Fig. 7c). Custom annotations were built in to review variants in CPGs and therapeutically actionable genes. We flagged compound events that potentially caused biallelic disruption of a gene. The interpretation history was displayed for recurrent deleterious or benign variants previously classified in other cases. Lastly, a knowledge-based prioritization tool was designed to automatically prioritize variants on the basis of these annotations. Short-listed variants were displayed in the variants of interest tab for classification and reporting.

### Interpretation of somatic and germline variants from the cancer panel

**Extended Data Fig. 8:**
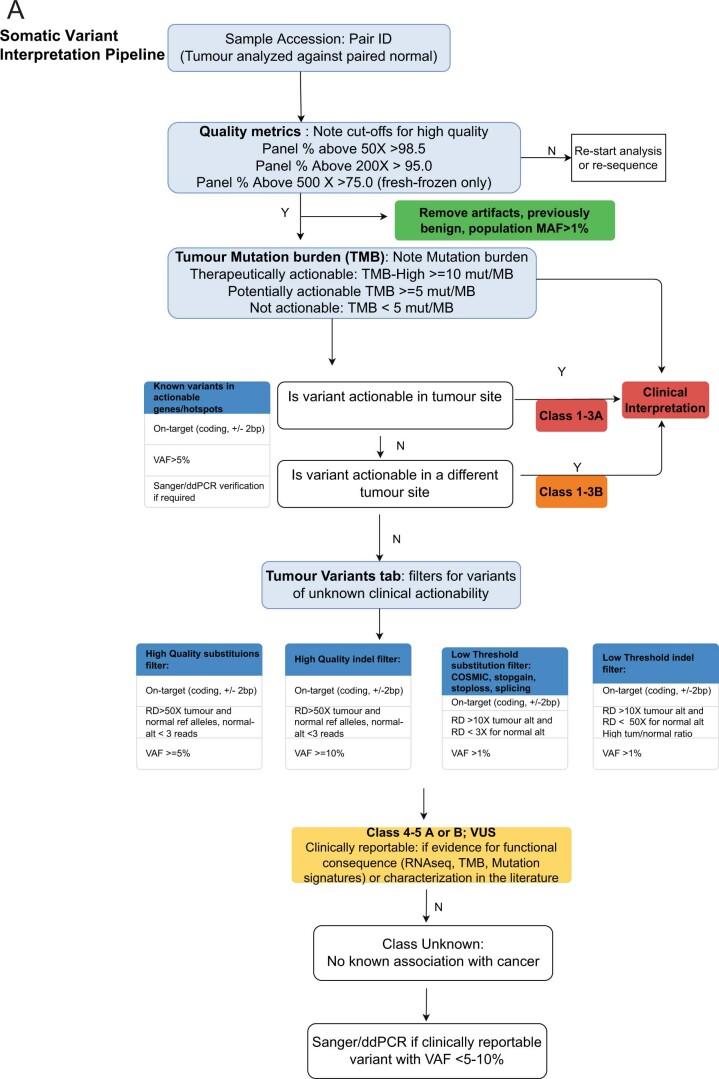
Somatic variant interpretation pipeline. Somatic variant interpretation scheme (Class I-V) is further detailed in Extended Data Fig. 4. VUS – clinical actionability of this variant is currently unknown; however, the variant may be biologically relevant in the patient’s tumor type. Unknown – no known association of this variant with cancer.

Small indels in clinically actionable genes were confirmed by Sanger sequencing or ddPCR if they met requirements for verification by these orthogonal methods. Alamut Visual (Interactive Biosoftware) was used to predict the potential splicing effects of variants found ±10 bp with respect to intron–exon junctions. Predictions were investigated in matched tumor-derived RNA, when available (Extended Data Fig. 8). Alamut parameters were as follows: synonymous variants, 3′- and 5′-UTR variants, and intronic variants (±10 bp) were reported if known to be pathogenic in ClinVar, if predicted to disrupt splicing as indicated by in silico programs (SpliceSiteFinder, MaxEntScan, NNSPLICE, GeneSplice) or if predicted to disrupt translation initiation. At least three programs were required to generate a prediction score of >30% each or a combined score of 100% for an indel to be considered disruptive. Variants beyond ±10 bp were not reported unless known to be pathogenic in ClinVar or the literature.

**Extended Data Fig. 9:**
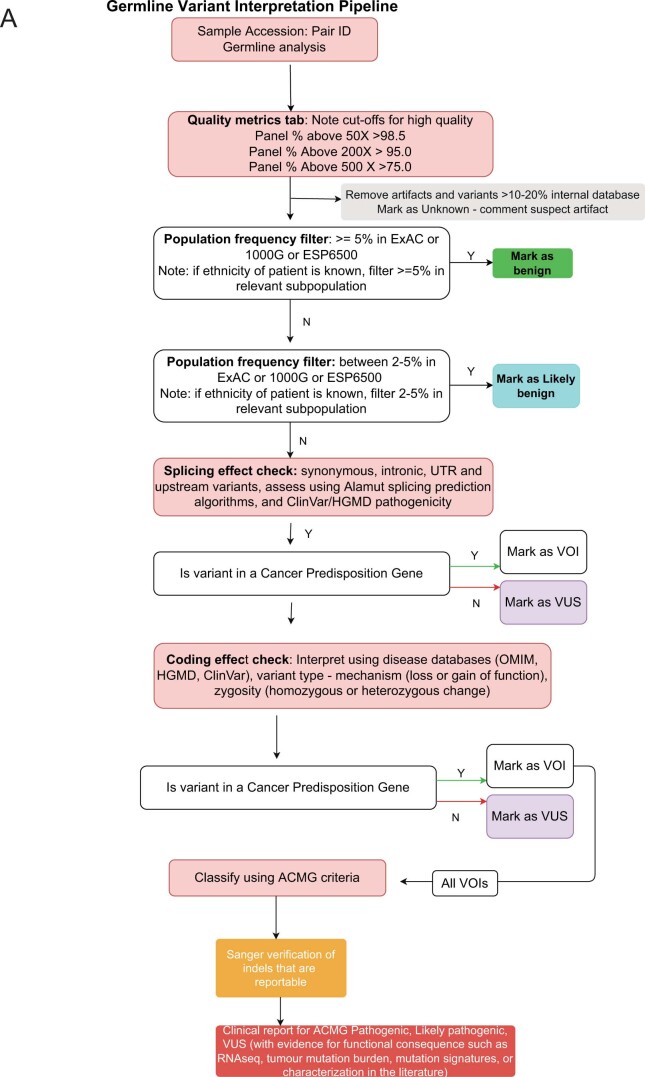
Germline variant interpretation pipeline. Abbreviations: VOI – variant of interest; VUS – variant of uncertain significance; ACMG – American College of Medical Genetics; HGMD – Human Genetic Mutation Database; UTR – untranslated region.

Somatic variants were classified as noted above (Extended Data Figs. 4, 6 and 8). Germline variants were classified using ACMG criteria^66^ with reference to recent ClinGen criteria^67^. Germline variants found in the general population at a frequency of >5% were classified as benign, and those found at a frequency of between 2–5% were classified as likely benign. LP/P variants, as well as variants of uncertain significance with supporting functional evidence, were reported. This analysis included functional characterization of the variant by other published studies, RNA-seq of the patient’s tumor showing aberrant splicing or loss of expression, high TMB or mutational signatures detected in the tumor due to variants in MMR or HRD genes (Extended Data Fig. 9 and Supplementary Table 12).

### RNA extraction and sequencing

Tumor samples were disrupted with an electric OMNI homogenizer with a disposable probe and processed using the Qiagen RNeasy Micro tissue extraction kit, with a maximum starting input of 1–5 mg (usually 3 mg). Total RNA quality was assessed using an Agilent Bioanalyzer 2100 RNA Nano chip. Concentration was measured by Qubit RNA HS assay on a Qubit fluorometer (ThermoFisher). RNA-seq library preparation was performed following the NEB NEBNext Ultra II Directional RNA Library Preparation protocol. In brief, 400 ng of total RNA was used as input, enriched for poly(A) mRNA, fragmented to a size of 200–300 bases for 4 min at 94 °C, converted to double-stranded cDNA, end-repaired and adenylated at the 3′ end to create an overhang A, allowing for ligation of Illumina adaptors with an overhang T. Library fragments were amplified under the following conditions: initial denaturation at 98 °C for 30 s; 15 cycles of 98 °C for 10 s, 65 °C for 75 s ; and extension for 5 min at 65 °C. Samples were amplified with different barcoded adaptors, enabling multiplex sequencing. One microliter of the library was loaded on a Bioanalyzer 2100 DNA High-Sensitivity chip to check for size. Libraries were quantified by qPCR using the Kapa Library Quantification Illumina/ABI Prism protocol (Kapa Biosystems) and then sequenced on a High-Throughput Run Mode flow cell with V4 sequencing chemistry on an Illumina HiSeq 2500 using 125-bp reads.

### Fusion detection

Fusion transcripts were detected by integrating multiple fusion detection algorithms (STAR-Fusion, Chimerascan, Mapsplice and deFuse), removing normal artifacts and validating the remaining fusions (F.F. et al., unpublished data). Following initial detection, candidate fusions were amalgamated, formatted and annotated using a common set of gene models (GTF file format). This standard format includes coordinates, strand and, if the breakpoint ends are within genes, gene names and locations, splice donor/acceptor sites and the exonic locations of the breakpoint ends. Potential read-through events and those involving microRNA, small nucleolar RNA, ribosomal and mitochondrial genes were removed. Each candidate fusion was reconstructed using 200 bp from the 5′ and 3′ break point ends.

To remove common artifacts, we aligned normal transcriptomes (1,277 GTEx samples from 43 tissues) to the candidate reconstructed chimeric transcripts (STAR v2.4.2a). Reads were considered to be aligned to a reconstructed chimeric transcript if they had a minimum overlap of 10 bp and were a perfect match. Chimeric transcripts with more than three reads from more than three GTEx samples were removed. The remaining candidate fusions were brought forward for validation.

Validation of the candidate fusions was performed by both local and genomic realignment. For local realignment, reads were locally realigned in single-end mode against each chimeric transcript remaining from the previous steps (STAR v2.4.2a). Aligned reads were extracted using samtools and used to reconstruct the sequence spanning the breakpoint. Validation of the breakpoint by reconstruction was achieved through two methods. One method used de novo assembly of the candidate reads with Abyss 2.1.0 using iteration of different *k*-mers. Contigs generated by Abyss were compared to the reconstructed chimeric transcript using BLAST. Fusions were considered validated if they had at least 3 bp of overlap with at most one mismatched base. The second method used was to create an artificial 5-bp gap on the candidate chimeric transcript at the breakpoint and use Gapfiller^68^ to close the artificially created scaffold between the two breakpoint ends. Reads that were previously selected as potential candidate reads of the transcript were used as input and aligned against the artificial scaffolds for each scaffold end using Bowtie (if shorter than 50 bp) or BWA (if longer than 50 bp). Reads aligned onto the scaffold were then split into shorter *k*-mers (85% of read) and used to iteratively fill the artificial gap from the left and right edges, one base at a time. Each base of the gap was considered filled if it was covered by at least two *k*-mers. A candidate fusion was considered validated if its gap was closed with at least 3 bp of overlap achieved on both the left and right edges of the artificial gap and the total length of the gap-filled sequence and the starting artificial chimeric transcript did not differ by more than 1 bp. For validation by genomic realignment, fusion chimeric transcripts were aligned to the reference genome (using BLAST, with DUST filtering for low-complexity regions). Chimeric transcripts aligning to low-complexity regions were flagged. Reconstructed chimeric transcripts aligning with more than 50 bp with 100% identity to other locations in the genome were considered to be misalignments. Lastly, for BLAST alignments spanning the breakpoint of the chimeric transcript, the difference between the end of the 5′ gene alignment and the breakpoint position and the difference between the start of the 3′ gene alignment and the breakpoint position were computed. The candidate fusion was flagged as an alignment artifact if the maximum of the two differences was greater than 10 bases with >99% identity.

Fusions were then scored on the basis of technical and biological features, to aid in interpretation. These features included, for example:

- Location of the breakpoints: the probability of a fusion being validated increased when both breakpoints were in a coding sequence.
- Exonic location of the breakpoint: having both breakpoints on a splice junction increased the probability of a fusion being real.
- Donor/acceptor site: the major canonical splice pattern GT–AG is more likely to be found in true fusions than the minor canonical (GC–AG and AT–AC) or noncanonical sequences.
- Frame: the predicted effect of the fusion was estimated using the Gene Rearrangement AnalySiS tool (https://github.com/cancerit/grass). Driver fusions are typically in frame.

### Processing of WGS data

Tumors and matched non-neoplastic samples were sequenced on an Illumina HiSeq X. The target depth was 30× for normal samples and 30× or 60× for tumors. Actual depths achieved were as follows: normal samples, average depth of 38.83× (range, 24.33× to 90.54×); tumors, average depth of 49.23× (range, 26.55× to 125.24×); 4.6% of samples were below the 30× target depth. In total, 569 genomes (272 non-neoplastic and 297 tumor) from 238 patients were analyzed. FASTQ files were aligned using BWA-MEM (v0.78) and PCR duplicates were marked with Sambamba (v0.7.0), with indel realignment (GATK v3.8) and base quality recalibration using GATK (v4.1.3).

### Whole-genome somatic variant calling

Somatic substitution and indel calling was performed with Mutect2 (GATK v4.1.3). Structural variant calling was performed using Delly (v0.7.1) and the GRIDSS-PURPLE-LINX container (https://github.com/hartwigmedical/gridss-purple-linx); Delly calls with at least four discordant read pairs were kept. All somatic variants (substitutions, indels and structural variants) were filtered for quality control as previously described^11^. In brief, we required a minimum depth of 10× in the tumor and normal samples with no reads supporting the variant in the normal sample. We removed variants found in a panel of non-neoplastic samples (*n* = 133), analyzed using the same methods, and those that failed at least two of the four cutoffs for nonunique mapping (<70% of reads at the locus mapping uniquely), multiply mapping clusters (seen in tumor and matched normal samples), excessively high mapping depth (compared to the average for the normal chromosome) and presence in low-complexity regions (DUST score of >60). Samples were removed from use in TMB or structural variant burden calculations if artifacts were suspected. One such example would be an artificially high TMB resulting from the proband’s blood being obtained after transplantation^60^.

Structural variants were first filtered to remove any variants previously discovered by cancer panel analysis. Breakpoint ends were categorized as exonic or intronic, annotated as being within an oncogene or tumor suppressor or categorized as being within a promoter region or intergenic. Structural variants were grouped by breakpoint ends (promoter–promoter, exon–exon, intron–exon, exon–intron, etc.) and then underwent interpretation. Breakpoints were assessed to determine whether they disrupted a functional or regulatory domain (UniProtKB). When available, RNA-seq data provided supporting evidence for over- or underexpression caused by the disruption.

### Cancer panel TMB calculation

TMB was calculated from the count of variants detected from the panel with a VAF of 10% or higher. The count was divided by the panel target size (3.012823 Mb), which included the exonic regions of the panel ± 10 bp for intron–exon junctions, to give a value in mutations per Mb. TMB values of ≥5 were considered actionable on the basis of trial eligibility (NCT02992964).

### Exome TMB calculation

TMB was calculated from the count of variants detected in the exome, captured with the Agilent SureSelect v4 kit, intersected with the cancer panel intervals. This count was divided by the cancer panel target size, producing an in silico cancer panel TMB from the exome.

### Whole-genome TMB calculation

TMB was calculated for substitutions and structural variants. The count of variants detected from WGS was divided by the size of the reference genome (2,897.310462 Mb).

### Finding agreement between whole-genome and cancer panel TMB

One difference between the panel and WGS was the limit of detection. The panel had an average depth of 1,100×, whereas the depth of WGS was closer to 38×. Supplementary Table 13 shows the ability of WGS to detect variants using the panel as a truth set (*n* = 170 tumors). WGS had a limited ability to detect variants of low VAF compared to the panel (5% limit of detection).

**Extended Data Fig. 10:**
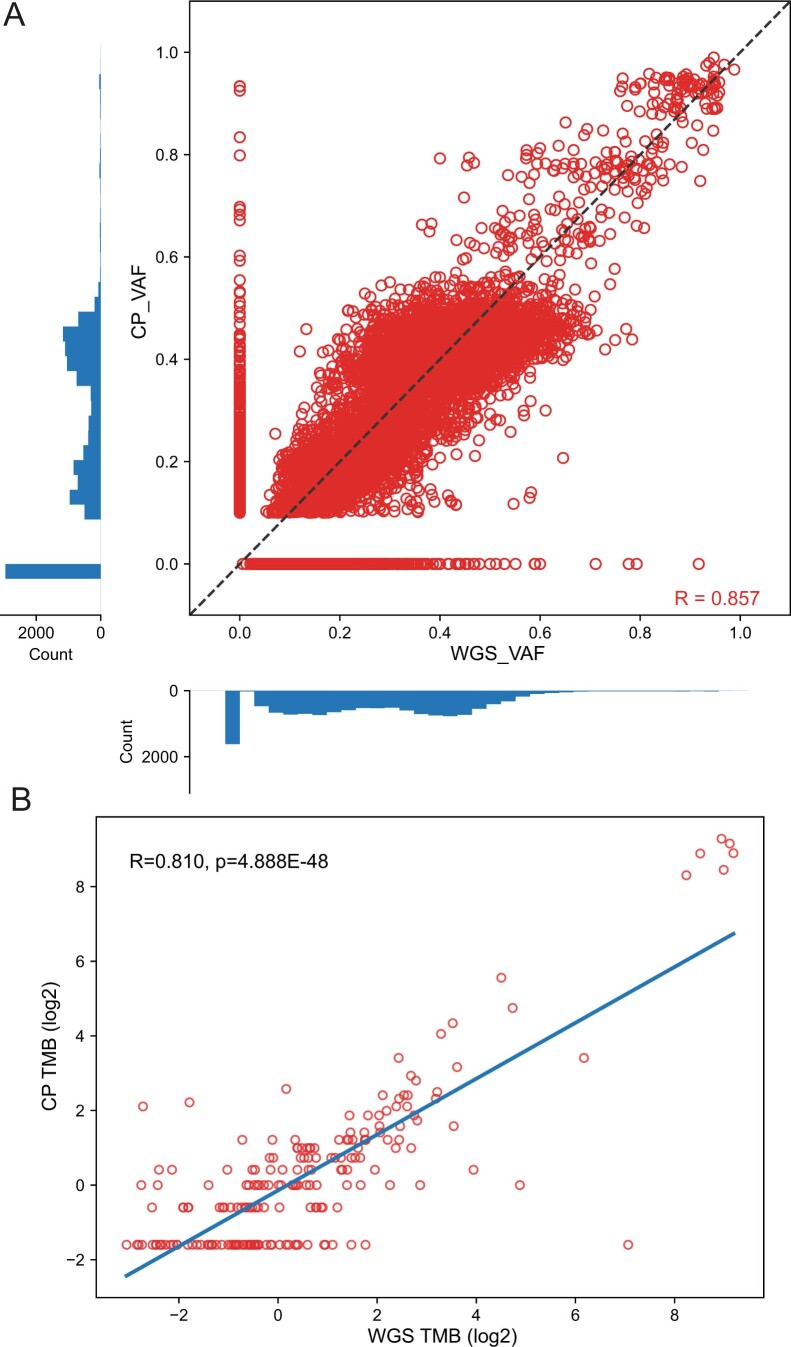
Agreement between whole genome and cancer panel tumor mutation burden. **(a)** Scatter plot of variant allele fraction from variants discovered on cancer panel target regions from 242 tumors from 193 patients detected on both cancer panel sequencing and whole genome sequencing. Axis plots are count histograms of variants at certain variant allele fraction bins. Variants discovered from one platform, but not the other, have the NULL value replaced with zero to allow plotting of the point, but these variants were not used within the correlation calculation. (**b)** Correlation of cancer panel tumor mutation burden to whole genome sequencing tumor mutation burden across 201 tumor samples. Only samples where cancer panel tumor mutation burden was greater than zero are plotted due to log conversion. Line indicates linear regression fit of these samples, Pearson (two sided) correlation R = 0.810, p = 4.888E^−48^. The Pearson (two sided) correlation and significance of non-log transformed data, including cancer panel samples with TMB of zero (303 tumor samples) was R = 0.956, p = 6.281E^−149^. Source data

These approaches were more concordant when the panel ‘truth set’ was restricted to variants with ≥10% VAF. Extended Data Fig. 10a shows that the variants detected in common were largely found at equivalent VAF but that there were still some private variants. These private variants included (1) somatic variants excluded from the cancer panel output owing to the low, yet unacceptable, number of alternative reads in the normal sample (due to the high depth of sequencing of the panel) not found in the normal sample by WGS and (2) true somatic variants excluded from the WGS output due to a few low-quality reads in the region, while the higher depth of sequencing in the cancer panel at these regions reduced the ratio of low-quality reads.

We observed a tight correlation between panel and WGS TMB (Extended Data Fig. 10b). We analyzed the difference in TMB for 170 individual samples (Supplementary Table 14). At VAF of ≥10%, WGS and the panel produced highly similar TMB. The average difference in TMB between the platforms was 0.42 mutations per Mb for samples with a TMB of <5 mutations per Mb (not deemed actionable), accounting for 91.2% of the 170 samples. For samples with 5–10 mutations per Mb, the average difference was 2.08 mutations per Mb, and, for samples with >10 mutations per Mb, a larger average difference was found (15 mutations per Mb), accounting for only 3.5% of the samples.

A limitation of the panel was the resolution of the TMB that could be called (~0.33 mutations per Mb for each variant on target). As shown in the top subplot of Extended Data Fig. 10a, the majority of samples had a negative value (that is, larger WGS TMB). If a tumor has a burden of 0.15 mutations per Mb and the resolution of the cancer panel is 0.33 mutations per Mb, a single mutation may not be in the target space, yielding a TMB of >0. Hence, some low-TMB samples had a negative difference between the cancer panel and WGS TMB.

### Copy number analysis

Copy number variants (CNVs) for cancer panel samples were called using CNVKit (0.9.4)^69^. Samples were compared to a reference of non-neoplastic tissues from males and females using CNVKit (*n* = 83). Sex-specific copy number normal reference modules were built from a series of ten male and ten female blood samples for NxClinical. Sex-matched patient samples were processed against this reference to generate copy number calls in NxClinical. The average bin size was set to 100 bp on target because of the high depth of the panel data; bin sizes for off-target regions were determined by CNVKit/NxClinical for genomic accessible regions using default settings. Mainly centromeres, telomeres and highly repetitive regions were excluded from the accessible regions. Bins with reference log_2_-transformed read depth of less than −5 or a spread of read depth larger than 1.0, predefined by CNVKit, were excluded for all sample analyses. Segments were called using circular binary segmentation. Normal CNV was defined as a log_2_-transformed ratio of 0 ± 0.2. Gains were defined as a log_2_-transformed ratio of >0.5, while losses were defined as a log_2_-transformed ratio of >−1.0. CNVs that included or disrupted a clinically actionable gene were classified according to the somatic variant interpretation scheme (see above). A decision tree was used to filter/prioritize CNVs for actionable gene targets (Supplementary Table 15).

The following steps were taken in NxClinical:

1. Removal of recurrent technical artifacts and common population variants (Database of Genomic Variants) with at least 95% reciprocal overlap
2. Prioritization of genic variants from actionable genes (Supplementary Table 15)
3. Review of losses encompassing tumor suppressors and gains/amplifications involving oncogenes
4. Review of whether the variant was a second hit to a mutation
5. Review of regions of LOH of >10 Mb involving actionable genes

The following steps were taken in CNVKit:

1. CNV variant calls were extracted for targeted, actionable genes, separated into ‘amplification’ or ‘deletion’ events and then filtered to ensure they were (1) focal (<5 Mb in length) and (2) true deletions (log_2_(ratio) < −2.0 for homozygous deletions and log_2_(ratio) < −0.85 for heterozygous deletions) and amplifications (log_2_(ratio) > 0.85 for high gains with greater than four copies and log_2_(ratio) > 2.3 for amplifications with greater than ten copies).
2. For cases with a germline or somatic deleterious substitution/indel in a tumor-suppressor gene, CNVs were analyzed to identify second hits in the same gene, either focal deletions (<5 Mb) or variants involving sizable segments or whole chromosomes.

All events passing cutoffs were visually inspected in IGV or NxClinical to ensure they were correctly sized and had sufficient copies as evidence for the CNV genotype.

### Extraction of SBS3 mutational signature

De novo extraction of SBS3 was performed using all somatic substitutions from 290 WGS samples, using the full cohort. One hundred mutations contributing to SBS3 were required to have a positive call. SigProfilerExtractor (v1.1) was used in two steps, as described previously^15^. The extracted signatures were compared to COSMIC Mutational Signatures v3.2–March 2021.

### DNA repair pathway analysis

Samples were divided into ‘HR germline’ (germline variants affecting the HR pathway; *n* = 20, 5 of which had multiple sequential samples) and ‘HR somatic’ (somatic HR variants; *n* = 20; 5 of which had multiple samples) (Supplementary Table 8). Each sample was also analyzed for SNVs, indels and copy number changes in HR pathway genes. In eight samples with biallelic variants, the first hit was germline followed by LOH or a second variant affecting the same gene in the tumor. In 13 samples with biallelic variants, both hits were somatic. In the ‘HR germline’ group, seven samples had biallelic mutations. In the ‘HR somatic’ group, 12 samples had biallelic variants. Fisher’s exact test of independence was used to determine whether SBS3 was significantly different between the PCAWG dataset and the three KiCS cohort subsets (‘non-HR’, ‘HR somatic’ and ‘HR germline’). Fisher’s exact test was performed in a pairwise fashion (that is, one test for each possible pair of datasets; Fig. 3b).

### Tumor evolution analysis

Thirty-eight patients had multiple neoplasms of the same histology in the same individual (Supplementary Table 10). Driver substitutions, indels and copy number changes were identified and tracked across subsequent relapses, progression samples or primary–metastatic pairs. Substitutions and indels were included in the analysis at any VAF (lowest 1.6%). Copy number changes were detectable in tumors with cellularity of at least 20–25%. Samples with ‘normal’ copy number profiles and TMB = 0 were suspected to have low tumor cellularity and were not included in this analysis. Variants were classified into (1) new therapeutically actionable drivers detected in the sample; (2) previously detected drivers absent in the sample, indicating displacement of the tumor clone and loss of a therapeutic target; (3) driver variants for which the VAF increased in the sample by >20%, indicating an expanded clone; (4) driver variants for which the VAF decreased by at least 20%, indicating a diminished clone; and (5) new drivers detected in the sample (not therapeutically actionable). Samples containing variants that could be classified in group 1 or 2 were counted as having a change in therapeutic actionability across serial sampling.

Pathology reports were reviewed before sequencing, and efforts were made to use samples/sections that did not have substantial necrosis or low tumor content (Extended Data Fig. 2b). As samples run on the panel were sequenced at high depth, it was possible to obtain reliable data even from samples with lower purity.

To show clonal evolution of an embryonal rhabdomyosarcoma in an individual with constitutional neurofibromatosis-1 (KiCS 15; Extended Data Fig. 5a), a mutation‐based phylogeny was created (Treeomics v1.8.1)^70^. Drivers in the trunk and branches were determined by WGS. We then used Pyclone-VI^71^ and PairTree^72^ clonal and phylogenetic analyses to develop a pipeline for all KiCS WGS data. For each case, PyClone-VI (a Bayesian method) inferred clonal populations, incorporating copy number and purity, and PairTree then reconstructed phylogenies and estimated cancer cell fraction (Fig. 6b and Extended Data Fig. 5). We compared pairs of initial primary tumors with metastases or relapses in multisample cases and then determined the proportion of mutations unique to the initial primary sample. Pairs in which >75% of the initial mutations shared^29^ were deemed linear.

### Statistics and reproducibility

No statistical method was used to predetermine sample size. The experiments were not randomized. The investigators were not blinded to allocation during experiments and outcome assessment. Individual analyses presented above, and the associated figures, include details on statistical methods and exclusions. Participant exclusions are presented in Fig. 1. None of the statistical tests used in this study required the assumption of normality or the assumption of equal variance.

### Reporting summary

Further information on research design is available in the Nature Portfolio Reporting Summary linked to this article.

## Supplementary information

Reporting Summary

Supplementary Tables Supplementary Tables 1–15.

## Data Availability

DNA and RNA sequencing data that support the findings of this study have been deposited in the European Genome-phenome Archive under accession codes EGAS00001006034 for RNA, EGAS00001006610 for DNA from WGS and EGAS00001006642 for DNA sequencing from the comprehensive cancer panel. All data not presented here that support the findings of this study are available from the corresponding author on request. Mutational signature data for PCAWG are publicly accessible at https://www.synapse.org/#!Synapse:syn11726602. Source data are provided with this paper.

## Source data

Source Data Fig. 2 Statistical source data.
Source Data Fig. 3 Statistical source data.
Source Data Fig. 5 Statistical source data.
Source Data Fig. 6 Statistical source data.
Source Data Extended Data Fig. 1 Statistical source data.
Source Data Extended Data Fig. 1 Statistical source data.
Source Data Extended Data Fig. 2 Statistical source data.
Source Data Extended Data Fig. 3 Statistical source data.
Source Data Extended Data Fig. 10 Statistical source data.
Source Data Extended Data Fig. 10 Statistical source data.

## Code availability

Custom code described in this study is available at https://github.com/shlienlab.
