# Supplementary material for: The clinical utility of integrative genomics in childhood cancer extends beyond targetable mutations
Source: Nat Cancer. 2022 Dec 30;4(2):203–21. doi: 10.1038/s43018-022-00474-y (PMC9970873; doi:10.1038/s43018-022-00474-y)
Supplement: Supplementary file 1 — Reporting Summary [file 43018_2022_474_MOESM1_ESM.pdf]

nature portfolio

Corresponding author(s): David Malkin and Adam Shlien

Last updated by author(s): Oct 24, 2022

Reporting Summary

Nature Portfolio wishes to improve the reproducibility of the work that we publish. This form provides structure for consistency and transparency in reporting. For further information on Nature Portfolio policies, see our Editorial Policies and the Editorial Policy Checklist.

Statistics

For all statistical analyses, confirm that the following items are present in the figure legend, table legend, main text, or Methods section.

n/a	Confirmed	
☐	☒	The exact sample size (*n*) for each experimental group/condition, given as a discrete number and unit of measurement
☐	☒	A statement on whether measurements were taken from distinct samples or whether the same sample was measured repeatedly
☐	☒	The statistical test(s) used AND whether they are one- or two-sided<br>*Only common tests should be described solely by name; describe more complex techniques in the Methods section.*
☐	☒	A description of all covariates tested
☒	☐	A description of any assumptions or corrections, such as tests of normality and adjustment for multiple comparisons
☐	☒	A full description of the statistical parameters including central tendency (e.g. means) or other basic estimates (e.g. regression coefficient) AND variation (e.g. standard deviation) or associated estimates of uncertainty (e.g. confidence intervals)
☐	☒	For null hypothesis testing, the test statistic (e.g. *F*, *t*, *r*) with confidence intervals, effect sizes, degrees of freedom and *P* value noted<br>*Give P values as exact values whenever suitable.*
☒	☐	For Bayesian analysis, information on the choice of priors and Markov chain Monte Carlo settings
☒	☐	For hierarchical and complex designs, identification of the appropriate level for tests and full reporting of outcomes
☒	☐	Estimates of effect sizes (e.g. Cohen's *d*, Pearson's *r*), indicating how they were calculated

Our web collection on statistics for biologists contains articles on many of the points above.

Software and code

Policy information about availability of computer code

Data collection	Participant enrollment details, sample details, clinical data, and discussion summaries were recorded in REDCap version 11.0.5.
Data analysis	Mutational signature analysis was performed using SigProfilerExtractor v1.1 publicly accessible at https://github.com/AlexandrovLab/SigProfilerExtractor.<br>Cancer Panel: BWA-MEM (v0.7.15), Picard (v2.5.0), GATK (v3.6.0), Mutect (v1.1.4), CNVkit (v0.9.4), NxClinical (v5.0)<br>Whole Genome: BWA-MEM (v0.7.8), Sambamba (v0.7.0), GATK (v3.8), GATK (v4.1.3), Delly (v0.7.1), gridss-purple-linx(v1.3.2)<br>RNA: STAR (v2.4.2a), STAR-Fusion (v0.8.0), Chimerascan (v0.4.5), Mapsplice (v2.1.8), deFuse (v0.6.2)<br>https://github.com/shlienlab<br>Treeomics (v1.8.1)

For manuscripts utilizing custom algorithms or software that are central to the research but not yet described in published literature, software must be made available to editors and reviewers. We strongly encourage code deposition in a community repository (e.g. GitHub). See the Nature Portfolio guidelines for submitting code & software for further information.

Data

Policy information about availability of data

All manuscripts must include a data availability statement. This statement should provide the following information, where applicable:

- Accession codes, unique identifiers, or web links for publicly available datasets
- A description of any restrictions on data availability
- For clinical datasets or third party data, please ensure that the statement adheres to our policy

Data Availability: DNA and RNA sequencing data that support the findings of this study have been deposited in the European Genome-phenome Archive (EGA) under accession codes EGAS00001006034 for RNA, EGAS00001006610 for DNA from whole genome sequencing, and EGAS00001006642 for DNA sequencing from comprehensive cancer panel. De-identified clinical data may be requested from the KiCS Program via the corresponding author, subject to required approvals (ex. REB) and data sharing agreements. Mutational signature data for PCAWG is publicly accessible at https://www.synapse.org/#!Synapse:syn11726602.
Code Availability: Custom code described in this study is available at github.com/shlienlab

Human research participants

Policy information about studies involving human research participants and Sex and Gender in Research.

Reporting on sex and gender	Sex (male or female) was collected from the medical record and included in the clinical data for each participant (and can be found in supplemental tables S1 and S2). The cohort was composed of 134 females and 166 males. Sex was not used as a variable in study analyses, and study findings are not specific to one sex. Sex was taken into account in the interpretation of sex-chromosome-based genetic variants.
Population characteristics	The population is pediatric and young adult participants, both male and female, of diverse ethnic backgrounds, with a variety of oncologic diagnoses (the majority associated with poor prognosis, and a minority of rare tumors). A minority of patients had no current cancer diagnosis but had a significant prior or family history of cancer which suggested potential underlying genetic susceptibility. Age, self-reported sex, and self-reported ethnicity can be found in Tables S1 and S2 and Extended data figure 1.
Recruitment	Patients are referred by their primary oncology teams based on the following eligibility criteria for enrollment onto one of two streams. Entry point 1 (tumor + germline analysis): individuals with a 'difficult-to-cure cancer,' defined as any upfront metastatic, poor prognosis (predicted 5-year overall survival <50%), or relapsed tumor; a poorly characterized rare tumor; or patients for whom next generation sequencing could potentially answer a clinically relevant question which had not been adequately addressed by clinical testing. Entry point 2 (germline +/- tumor analysis): individuals with a strong suspicion for a cancer susceptibility syndrome based on personal or family cancer history, with negative targeted clinical testing. Some individuals declined to participate in the study, and reasons are detailed in Figure 1. This represents a small proportion (5%) and is not expected to impact the results of this study.
Ethics oversight	The Hospital for Sick Children Research Ethics Board provided ethical oversight. All participants (or substitute decision makers) provided informed consent to participate in the study. Participants did not receive compensation.

Note that full information on the approval of the study protocol must also be provided in the manuscript.

Field-specific reporting

Please select the one below that is the best fit for your research. If you are not sure, read the appropriate sections before making your selection.

☒ Life sciences ☐ Behavioural & social sciences ☐ Ecological, evolutionary & environmental sciences

For a reference copy of the document with all sections, see nature.com/documents/nr-reporting-summary-flat.pdf

Life sciences study design

All studies must disclose on these points even when the disclosure is negative.

Sample size	No sample size was predefined. Sample size was based on the number of enrolled patients with available data.
Data exclusions	Quality thresholds were established for cancer panel, RNAseq and WGS data, for both fresh frozen and FFPE samples as well as for blood, as described in the methods. Samples failing sequencing were replaced with new samples and when not available, were excluded.
Replication	Accuracy and reproducibility of variant calls made from the cancer panel were tested during its validation, as described in detail in the methods. Replicates were carried out in triplicate within a run and in triplicate between sequencing runs. Periodic repeats across machines were also carried out every 6 months to ensure machine comparability. All attempts at replication were successful.
Randomization	Randomization was not performed in this study. This study was a prospective, non-interventional cohort study and randomization was not relevant.

Blinding	Binding was not performed in this study as it was not relevant for this prospective, non-interventional cohort. This study did not perform an assessment of patient outcomes.

Reporting for specific materials, systems and methods

We require information from authors about some types of materials, experimental systems and methods used in many studies. Here, indicate whether each material, system or method listed is relevant to your study. If you are not sure if a list item applies to your research, read the appropriate section before selecting a response.

Materials & experimental systems

n/a	Involved in the study
☒	☐ Antibodies
☒	☐ Eukaryotic cell lines
☒	☐ Palaeontology and archaeology
☒	☐ Animals and other organisms
☒	☐ Clinical data
☒	☐ Dual use research of concern

Methods

n/a	Involved in the study
☒	☐ ChIP-seq
☒	☐ Flow cytometry
☒	☐ MRI-based neuroimaging
